# Synchronizing the Osteochondral Regeneration Process through Spatial Patterning of Stable and Hypertrophic Cartilage Organoids

**DOI:** 10.1002/adma.202516189

**Published:** 2026-05-08

**Authors:** Liuqi Peng, Isaak Decoene, Hanna Svitina, Ioannis Papantoniou

**Affiliations:** ^1^ Prometheus the Translational Division of Skeletal Tissue Engineering Leuven R&D, KU Leuven, O&N1 Leuven Belgium; ^2^ Skeletal Biology and Engineering Research Center Department of Development and Regeneration KU Leuven, O&N1 Leuven Belgium

**Keywords:** assembloids, developmental engineering, induced pluripotent stem cells, organoids, osteochondral defect regeneration

## Abstract

Repairing deep osteochondral defects remains clinically challenging due to the intrinsic inability of articular cartilage to self‐repair and the need for integrated yet distinct regeneration of articular cartilage and subchondral bone. Here, we present a scaffold‐free, modular strategy that spatially bioassembles induced pluripotent stem cell (iPSC)‐derived chondrocytes (iChon) organoids with human periosteum‐derived cell (hPDC) organoids to engineer zonated osteochondral assembloids. The resulting iChon+hPDC assembloids exhibit intrinsic spatial organization, forming chondral‐ and osteo‐like zones with an intermediate interface without exogenous scaffolds. In vitro characterization confirmed layered glycosaminoglycan‐rich cartilage and collagen I‐rich osteo‐associated domains, with interface continuity emerging through self‐directed matrix organization. Upon implantation into full‐thickness osteochondral defects, iChon+hPDC assembloids promoted robust hyaline‐like cartilage repair, supported subchondral bone formation with ongoing repair/remodeling, and partially reestablished collagen fiber anisotropy. Protein‐level mapping further supported a surface‐associated cartilage phenotype and remodeling‐associated signatures in the deep compartment. Transcriptomic profiling revealed complementary biological programs, with iChon showing features suggestive of stable cartilage regulation and extracellular‐matrix remodeling competence, and hPDC showing a transient hypertrophic program associated with endochondral ossification. This work provides a scaffold‐free design framework for engineering zonated osteochondral implants through spatial assembly of lineage‐specific organoid modules, with translational potential for future osteochondral repair strategies.

## Graphical abstract

Zonated osteochondral assembloid implants are engineered through scaffold‐free spatial assembly of induced pluripotent stem cell (iPSC)‐derived chondrocyte organoids and periosteum‐derived osteogenic organoids. Implanted into full‐thickness rat osteochondral defects, they promote hyaline‐like cartilage repair, support subchondral bone formation with ongoing remodeling, and partially restore collagen anisotropy, providing a modular strategy for osteochondral implant engineering.

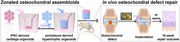

## 1 Introduction

Deep osteochondral defects pose a significant clinical challenge due to the limited regenerative capacity of articular cartilage and the structural complexity of the osteochondral unit [1, 2, 3, 4]. When left untreated, these defects often result in full‐thickness osteochondral degeneration, compromising subchondral bone and leading to joint dysfunction [5]. Current clinical treatments, such as microfracture and autologous chondrocyte implantation (ACI), often lead to the formation of mechanically inferior fibrocartilage [6, 7], whereas osteochondral allografts face limitations such as donor scarcity and immune rejection [8]. A recent meta‐analysis of 1,861 patients revealed long‐term failure rates of up to 38.1% for the regeneration of deep osteochondral defects in the knee using existing surgical interventions [9]. These results highlight the urgent need for regenerative strategies capable of restoring the hierarchical organization and functional integrity of native osteochondral tissue.

Tissue engineering (TE) strategies aim to address these limitations by combining cells with synthetic or natural biomaterials [10]. Recent clinical trials have validated the efficacy of engineered nasal chondrocyte‐based grafts for articular cartilage repair, demonstrating superior clinical outcomes compared to conventional clinical methods [11]. Early scaffold‐based approaches focused on seeding or encapsulating single‐cell suspensions like articular chondrocytes (ACs) or mesenchymal stromal cells (MSCs) into biodegradable polymers like polycaprolactone (PCL) [12, 13] or hydrogels (e.g., gelatin methacryloyl, hyaluronic acid) [14, 15]. However, these scaffold‐based systems face critical limitations, for example, rapid hydrogel degradation often precedes neotissue formation [16]. Additionally, stiff polymers like PCL can induce mechanical mismatch with native bone, causing stress shielding and poor integration [17]. Even advanced multiphasic scaffolds designed to mimic osteochondral gradients [18, 19, 20] suffer from interfacial delamination and poor cell retention under physiological loads, failing to achieve long‐term osteochondral functionality.

Recognizing these challenges, tissue engineers have drawn inspiration from embryonic joint development, where mesenchymal progenitors undergo condensation and differentiation to establish zonal cartilage–bone boundaries through cell–cell interactions and biomechanical signaling [21]. Translating this paradigm, the development of cartilage organoid‐based strategies has emerged as an alternative to traditional scaffolds [22]. These approaches use predifferentiated organoids as living building modules, harnessing the inherent capacity of specific cell types to produce near‐native extracellular matrix complexity and stimulate tissue integrations through active signaling. These zonal modules can be spatially arranged within biomaterials or 3D‐printed architectures to recapitulate osteochondral zonation. One study employed a bioprinting method using hMSC spheroids as modules ladened with xanthan gum‐alginate hydrogel [23], while another study engineered biphasic osteochondral constructs by positioning human adipose‐derived stem cell (hADSC) modules primed with TGF‐β3 (cartilage) or BMP‐2 (bone) within 3D‐printed microchambers [24]. However, these hybrid systems still depend on external materials (e.g., hydrogel carriers/bioinks or 3D‐printed holders/microchambers), which can leave residual fragments that disrupt native tissue integration and add material‐related complexity [25]. Additionally, MSC‐derived cartilage is prone to *RUNX2*‐mediated hypertrophy under mechanical stress [26], while primary ACs exhibit donor‐dependent variability and dedifferentiation during expansion [27], limiting their clinical applicability. Recent coculture strategies, such as combining ACs and MSCs in alginate hydrogels, have demonstrated enhanced cartilage stability and reduced hypertrophy in vivo [28]. Similarly, the direct hMSC–human articular chondrocytes (hAC) modules contact promotes hyaline differentiation and tissue integration [29]. These findings highlight the importance of integrating cartilage‐ and bone‐forming cell populations to achieve functional zonation. Nevertheless, the continued reliance on primary ACs introduces batch variability and scalability issues.

Induced pluripotent stem cells (iPSCs) provide a promising alternative for osteochondral regeneration, offering unlimited expansion, patient specificity, and the ability to generate stable hyaline cartilage resistant to hypertrophy [30]. Compared to MSC‐derived chondrocytes, iPSC‐derived chondrocytes (iChon) produce a collagen II‐rich extracellular matrix with minimal fibrotic or hypertrophic changes, demonstrating superior cartilage regeneration in preclinical models [31, 32]. Advances in stepwise differentiation protocols [33], 3D culture systems [34, 35], and genetic modifications such as CRISPR/Cas9‐mediated *COL2A1*–GFP knock‐in reporter iPSC line [36] have enhanced cartilage stability. Moreover, iPSC‐based implants have successfully restored cartilage in large animal models, reinforcing their translational potential [37, 38]. However, despite these advances, existing iPSC strategies have primarily focused on cartilage regeneration without addressing the concurrent need for subchondral bone formation. Additionally, most current iPSC‐based approaches rely on macromass, pellet cultures or self‐organized cartilage nodules, lack spatial control over zonal differentiation, limiting their ability to replicate the hierarchical organization of native osteochondral tissue.

To address these challenges, we developed a scaffold‐free zonated assembloid by integrating iChon‐derived cartilage organoids with hypertrophic cartilage organoids generated from human periosteum‐derived cells (hPDC) without relying on hydrogel carriers/bioinks or printed supporting architectures. Building on our previous work demonstrating the capacity of hPDC to support subchondral bone formation via endochondral ossification [39], this strategy leverages the self‐organizing potential of iChon organoids able to generate stable, hypertrophy‐resistant cartilage. By spatially arranging iChon and hPDC organoids to create an assembloid termed iChon+hPDC, our approach establishes a scaffold‐free, spatially organized living construct in which the overall architecture is not imposed by a passive external scaffold, but is encoded by the identity, spatial arrangement, and fusion behavior of the living modules themselves, with the interfacial transition emerging through self‐directed matrix organization. Compared to constructs based on hAC and hPDC assembloid (hAC+hPDC), the iChon+hPDC assembloid tended to display a more distinct zonal architecture and clearer compartmental stratification. Furthermore, to evaluate its therapeutic potential, we orthotopically implanted the engineered assembloids into deep osteochondral defects of knee joints and assessed their ability to integrate with host tissue and maintain zonal organization. This study explores the potential of zonated assembloids as a novel strategy for coordinated repair of articular cartilage and subchondral bone, addressing key challenges in joint defect repair.

## 2 Results

### 2.1 Differentiation and Characterization of iPSC‐Derived Chondrocyte Macromass (iChonMass)

To generate a scalable and phenotypically stable source of chondrocytes, we differentiated human iPSCs into cartilage‐like tissue using a previously established two‐step protocol involving mesodermal induction and chondrogenic maturation [40], as illustrated in Figure S1a. Morphological observations throughout the differentiation process showed a transition from the typical pluripotent stem cell colonies (Day 0) to an elongated loosely aggregated morphology (Day 3), progressing to compact aggregates on day 7. By day 28, visible cartilage‐like nodules had formed, which further matured into iChonMass with diameter of approximately 2–3 mm by week 8 (Figure S1b,S2a,b). Histological and gene expression analysis confirmed initiation and progression of chondrogenic differentiation. Safranin O/Fast Green (SafO/FG) staining revealed uniform deposition of glycosaminoglycans throughout the iChonMass, indicating homogenous cartilaginous extracellular matrix formation (Figure S2c). Gene expression analysis confirmed the chondrogenic differentiation through the upregulation of key chondrogenic markers. Notably, *SOX9* increased by 117‐fold by week 6 in comparison to week 0, while *ACAN* and *COL2A1* peaked at week 8 with approximately 13 354‐fold and 7859‐fold changes, respectively (Figure S2d). In contrast, *COL10A1* expression was downregulated at early stages and remained suppressed, indicating a lack of hypertrophic differentiation in the iChonMass.

To further evaluate phenotypic stability in vivo, iChonMass constructs were subcutaneously implanted into immunocompromised mice for 8 weeks (Figure S3a). NanoCT imaging with Hexabrix contrast revealed distinct cartilaginous regions surrounded by soft tissues, and 3D reconstructions confirmed the preservation of cartilage‐like morphology and a lack of mineralized structures within the explants (Figure S3b,c). Histological staining of the explants further validated the chondrogenic phenotype. Hematoxylin and eosin (H&E) staining showed dense extracellular matrix organization with no signs of fibrous or mineralized tissue formation (Figure S3d). Consistently, SafO/FG staining confirmed abundant proteoglycan deposition (Figure S3e), reinforcing the cartilage‐like characteristics even after 8 weeks in vivo. Together, these findings confirmed the successful differentiation of iPSCs into iChonMass which can be used as a robust source for articular‐like chondrocytes suitable for subsequent cartilaginous organoids formation.

### 2.2 Formation and Characterization of Organoids from iChon, hAC, and hPDC

**FIGURE 1:**
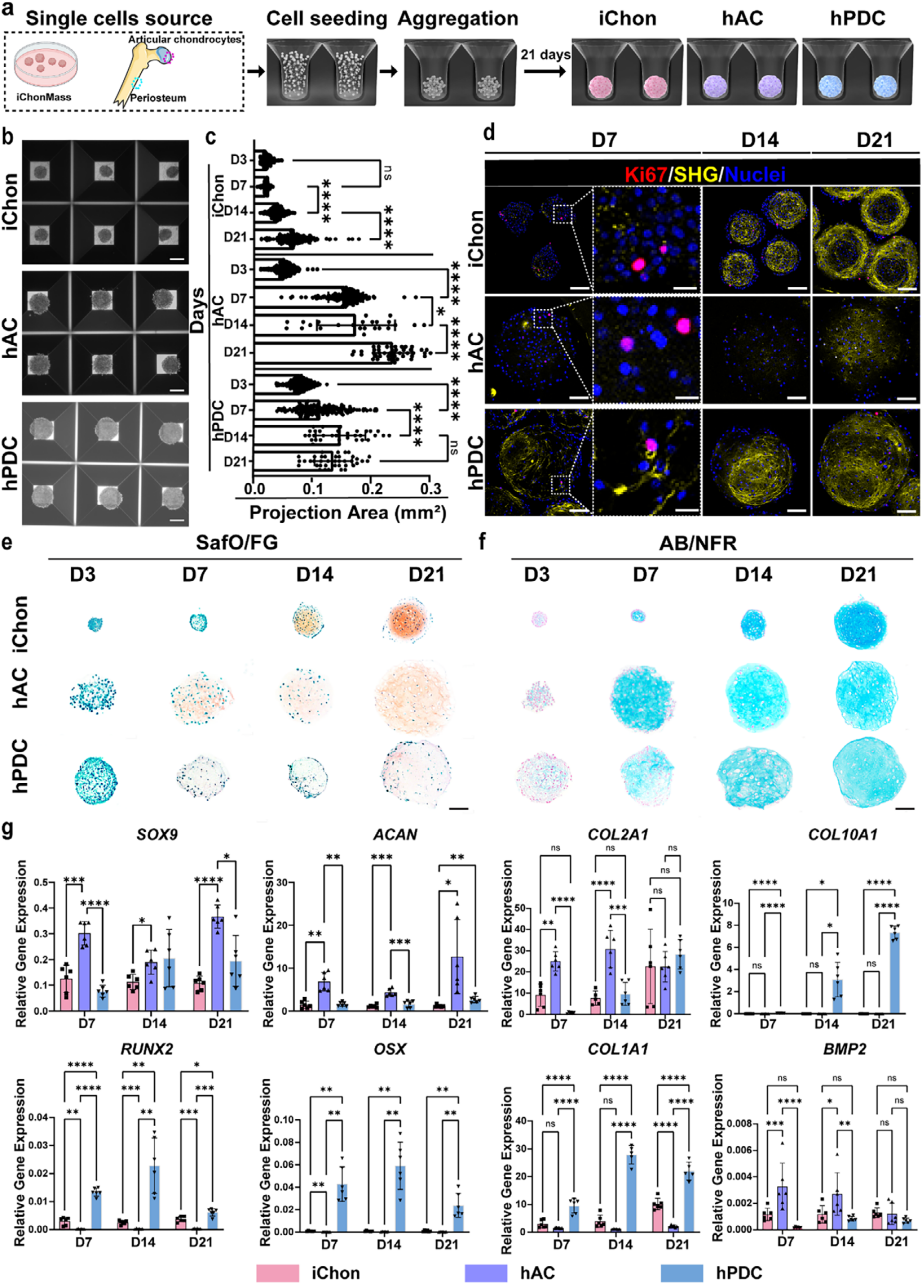
Formation and characterization of iChon, hAC, and hPDC organoids. (a) Schematic representation of the formation process of cartilaginous organoids from iPSC‐derived chondrocytes (iChon), human articular chondrocytes (hAC), and human periosteum‐derived cells (hPDC). (b) Representative bright‐field images of iChon, hAC, and hPDC organoids in AggreWell at day 7 (scale bar: 200 µm). (c) Quantification of organoid projection area over time (day 3, 7, 14, and 21) for all three groups. Data presented as mean ± standard deviation (SD); *n* > 20 organoids per group per time point, each dot represents one organoid; *p*‐values are calculated using one‐way analysis of variance (ANOVA) with Tukey's multiple comparisons test within each group across time. ns, not significant; *p < 0.05; **p < 0.01; ***p < 0.001; ****p < 0.0001. (d) Immunofluorescence staining for Ki67 (red), second harmonic generation signal (yellow), and nuclei (blue) at day 7, 14, 21 of different types of organoids. For the iChon group, panels are composed of stitched fields containing multiple organoids (scale bar: 100 µm). (e) Safranin O/Fast Green (SafO/FG) and (f) Alcian Blue/Nuclear Fast Red (AB/NFR) staining of organoids at day 7, 14, and 21 (scale bar: 100 µm). (g) Temporal gene expression profiles of chondrogenic (*COL2A1*, *SOX9*, *ACAN*), hypertrophic (*COL10A1*, *RUNX2*, *OSX*), and osteogenic (*COL1A1*, *BMP2*) markers normalized to *β‐actin*. Data presented as mean ± SD; *n* = 6 wells per group; *p*‐values are calculated using one‐way ANOVA followed by Tukey's multiple comparisons test. ns, not significant; *p < 0.05; **p < 0.01; ***p < 0.001; ****p < 0.0001.

In order to enable bioassembly of zonated osteochondral constructs, we generated size‐controlled cartilage organoids from three distinct cell populations. First, iChon single cells were derived by enzymatically dissociating mature iChonMass while hAC and hPDC were directly used as primary cells. Cartilage organoids were formed by seeding single cells in their respective chondrogenic medium into non‐adherent Aggrewell800 microwells. Cells underwent aggregation, condensation and differentiation over 21 days as shown in Figure 1a. Bright field imaging on day 7 showed that all three cartilage organoid types formed compact spherical structures, but with size differences (Figure 1b). Time course imaging from day 3 to day 21 showed progressive changes in organoid size and morphology of all three types (Figure S4). Quantitative analysis of organoid projection area over time revealed that all three groups exhibited a progressive increase in size from day 3 to day 21 (Figure 1c). However, iChon organoids maintained a smaller physical scale relative to hAC and hPDC throughout the culture period. At day 21, iChon reached an average area of 0.067 mm^2^, compared to 0.217 and 0.137 mm^2^ for hAC and hPDC, respectively (Figure 1c). These differences reflect distinct compaction and extracellular matrix (ECM) deposition dynamics among the three cell types.

Furthermore, immunofluorescence analysis and second harmonic generation (SHG) imaging were used to assess proliferation and collagen organization (Figure 1d and Figures S5, S6). Ki67 (marker of proliferation) staining revealed limited proliferative activity in iChon and hAC organoids across all timepoints, while hPDC organoids exhibited a peak in proliferation at day 7, followed by a marked decline. SHG imaging showed overall low collagen‐related SHG signal at day 7, with ridge‐traceable SHG fiber‐like features already detectable in hPDC organoids. By day 21, iChon organoids displayed increased peripheral SHG signal, whereas hPDC organoids showed stronger and asymmetrically distributed signals from early timepoints. In contrast, hAC organoids showed minimal SHG signal throughout the culture period. Additional SHG quantification using a fixed ridge‐based analysis pipeline is provided in Figures S7, S8 and Table S1. Histological evaluation further confirmed matrix accumulation. SafO/FG and Alcian Blue/Nuclear Fast Red (AB/NFR) staining revealed progressive sulfated glycosaminoglycans (GAGs) deposition in all groups (Figure 1e,f), with iChon and hAC organoids displaying more intense and uniform staining compared to hPDC, which exhibited lower GAG‐associated staining throughout differentiation.

In order to further evaluate gene expression profiles among the three types of organoids, we performed reverse transcription quantitative PCR (RT‐qPCR) analysis of key chondrogenic, hypertrophic, and osteogenic genes at days 7, 14, and 21 (Figure 1g). Among chondrogenic markers, *SOX9* expression was highest in hAC across all time points, with a significant difference compared to iChon and hPDC at day 21, while iChon and hPDC showed moderate expression levels with no clear upregulation trend over time. *ACAN* expression followed a similar pattern, with hAC consistently showing the highest levels, whereas iChon and hPDC remained lower throughout the time course. In contrast, *COL2A1* expression increased over time in both iChon and hPDC, with iChon displaying higher levels than hPDC at early time points. By day 21, *COL2A1* expression was comparable across all three groups, suggesting progressive matrix maturation in iChon and hPDC organoids. For hypertrophic and osteogenic markers, *COL10A1*, *RUNX2*, and *OSX* were markedly elevated in hPDC, indicating a shift toward hypertrophic phenotype. iChon and hAC maintained low expression of these markers, consistent with stable chondrogenic phenotype. *COL1A1* expression was also highest in hPDC across all time points, while *BMP2* remained at low levels in all groups without significant variation at day 21. These gene expression profiles further support the phenotypic divergence observed among the three organoid types, reinforcing the chondrogenic identity of iChon and hAC, and the osteogenic trends of hPDC. Taken together with the morphological and histological observations, these findings provide further rationale for the combinatorial use of iChon or hAC and hPDC in spatially organized osteochondral assembloid construct architecture.

**FIGURE 2:**
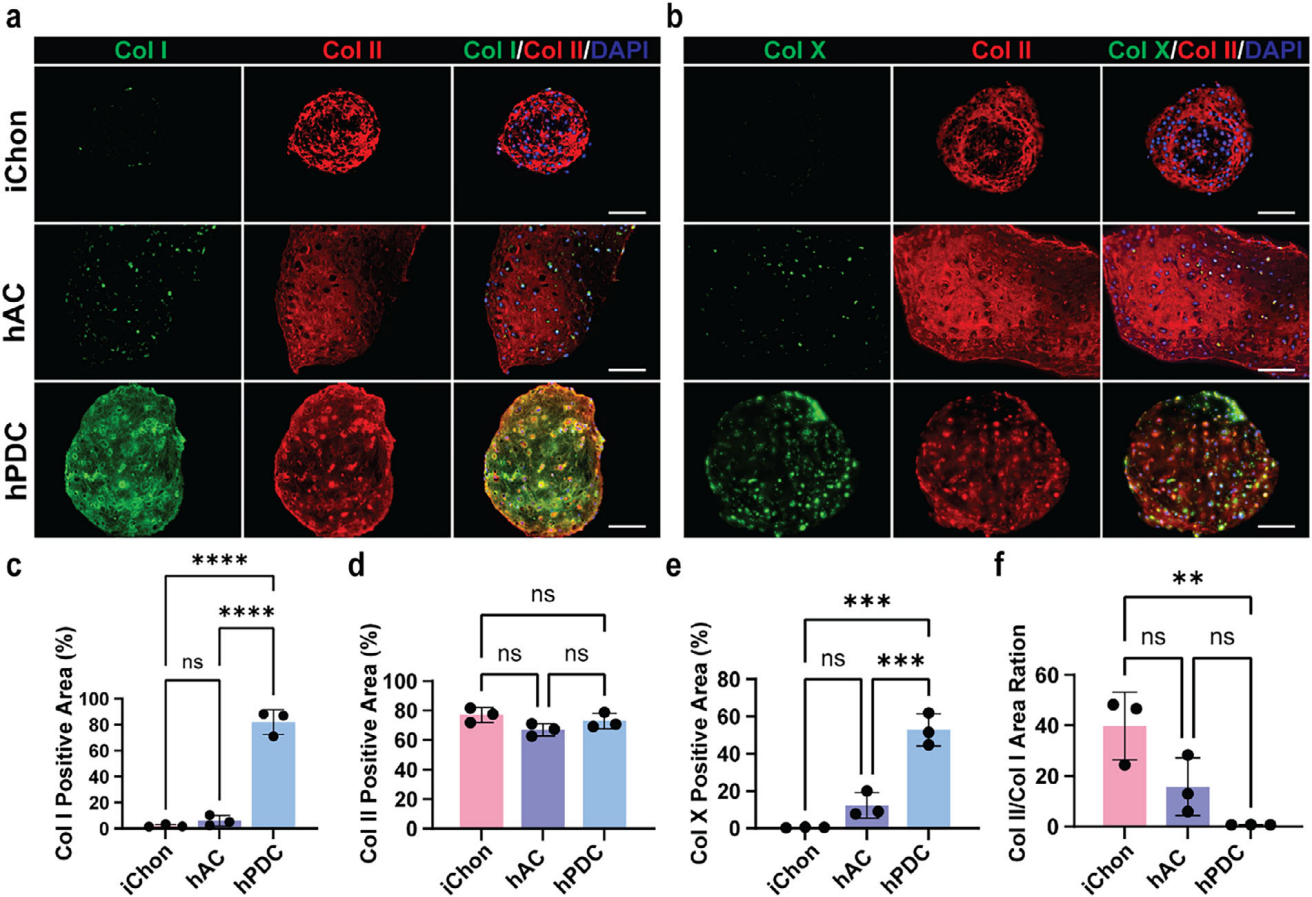
Molecular phenotyping and cartilage matrix maturity assessment. (a) Representative immunofluorescence images of Collagen Type I (Col I, green) and Collagen Type II (Col II, red) in three types of organoids at day 21 (scale bar: 100 µm). (b) Representative immunofluorescence images of Col II (red) and Collagen Type X (Col X, green) at day 21, highlighting the phenotypic stability of iChon and hAC versus the hypertrophic trend in hPDC organoids (scale bar: 100 µm). (c) Semiquantitative analysis of Col I, (d) Col II, and (e) Col X positive area (%) within the organoids at day 21. (f) Differentiation index calculated as the ratio of Col II to Col I positive area, representing the hyaline‐like matrix quality. Data presented as mean ± SD; *n* = 3 organoids per group; *p*‐values are calculated using one‐way ANOVA followed by Tukey's multiple comparisons test. ns, not significant; *p < 0.05; **p < 0.01; ***p < 0.001; ****p < 0.0001.

To further confirm phenotypic matrix profiles at the protein level, day 21 iChon, hAC, and hPDC organoids were immunostained for type II collagen (COL II) together with either type I collagen (COL I) or type X collagen (COL X) (Figure 2). All three organoid types displayed abundant COL II‐positive matrix, and quantitative analysis showed no significant difference in COL II‐positive area among groups (Figure 2a,d). In contrast, COL I deposition differed markedly between populations: iChon and hAC organoids exhibited low COL I signal, whereas hPDC organoids showed extensive COL I positivity throughout the tissue (Figure 2a), resulting in a significantly increased COL I‐positive area in hPDC compared with both iChon and hAC (Figure 2c). Similarly, COL X staining remained minimal in iChon and low in hAC, but was strongly enriched in hPDC organoids, with significantly higher COL X‐positive area compared with both cartilage‐derived organoids (Figure 2b,e). Consistent with these patterns, the COL II/COL I area ratio was numerically highest in iChon and lowest in hPDC, with a significant difference between iChon and hPDC, while hAC exhibited an intermediate ratio (Figure 2f). Collectively, these data demonstrate that iChon and hAC organoids form a COL II‐dominant cartilage‐like matrix, whereas hPDC organoids are characterized by pronounced COL I/COL X deposition, consistent with a hypertrophic cartilage‐like phenotype.

### 2.3 Sequential Bioassembly of Chondrogenic Organoids to Generate Zonated Osteochondral Assembloids

**FIGURE 3:**
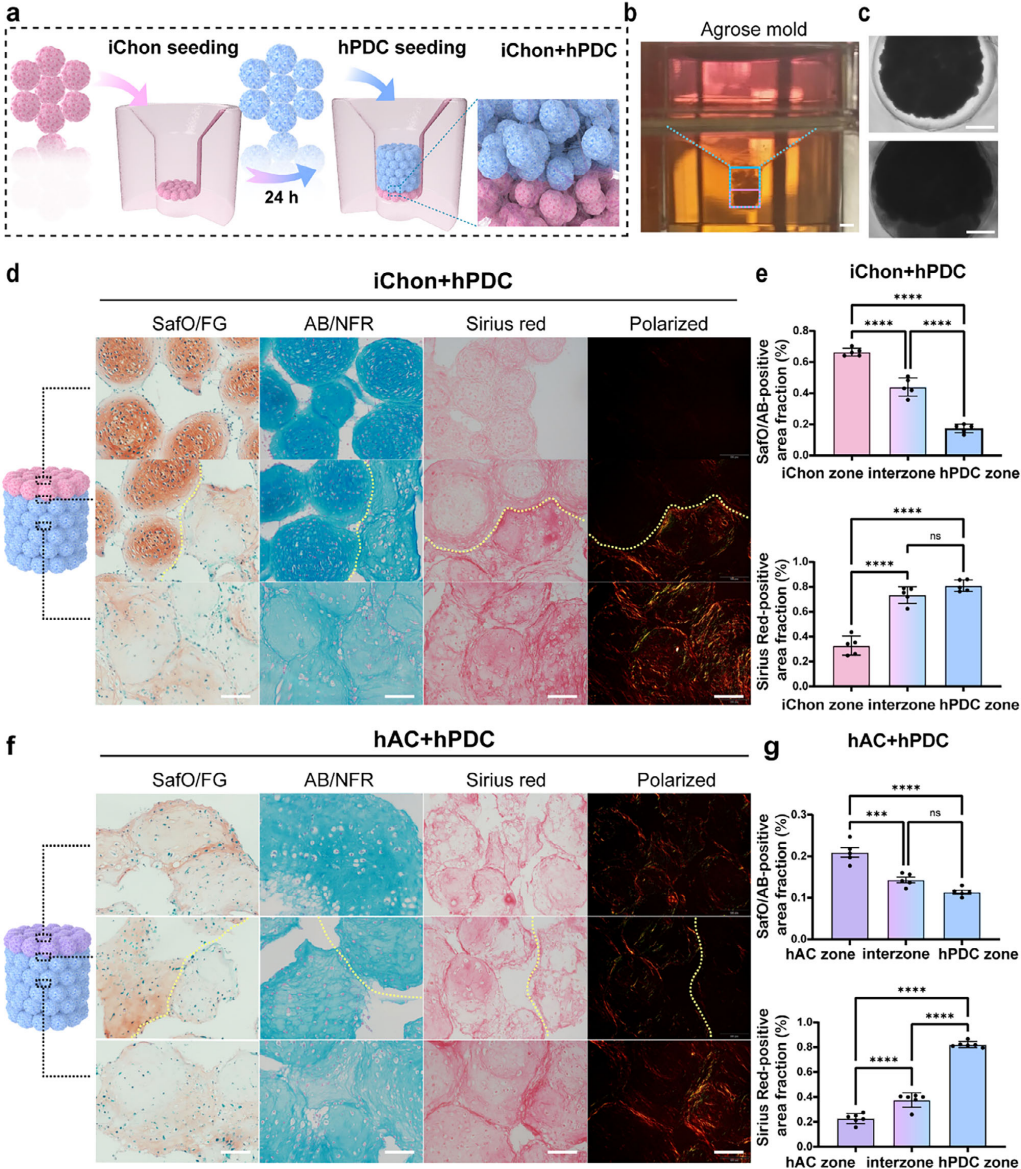
Modular bioassembly and characterization of zonated osteochondral assembloids. (a) Schematic of the modular bioassembly strategy for osteochondral assembloids. (b) Agarose mold designed to spatially organize chondral‐layer (bottom) and osteo‐layer (top) organoids (scale bar: 500 µm). (c) Bright‐field images of the stratified organoid layers post‐assembly (scale bar: 500 µm). (d) Histological evaluation of iChon+hPDC assembloids: SafO/FG, AB/NFR, and Sirius Red staining (scale bar: 100 µm) with polarized light imaging highlighting collagen organization (yellow dashed line shows the boundary of the osteochondral assembloids in iChon+hPDC and hAC+hPDC). (e) Semiquantification of glycosaminoglycans (GAG)‐associated staining readouts and Sirius Red‐based collagen staining readouts in iChon+hPDC assembloids. (f) Identical histological staining for hAC+hPDC assembloids (scale bar: 100 µm). (g) The corresponding semiquantitative analysis of GAG‐associated staining readouts and Sirius Red‐based collagen staining readouts in hAC+hPDC assembloids. Data presented as mean ± SD; *n* = 3 assembloids per group; *p*‐values are calculated using one‐way ANOVA followed by Tukey's multiple comparisons test. ns, not significant; ***p < 0.001; ****p < 0.0001.

To recapitulate the osteochondral tissue hierarchy, we developed a zonated bioassembly strategy utilizing agarose molds to spatially organize two distinct chondrogenic organoid populations: a chondral‐layer (using day 21 iChon or hAC organoids) and an osteo‐layer (using day 21 hPDC organoids). The chondral‐layer organoids (iChon or hAC) were first seeded and allowed to fuse for 24 h, followed by seeding of the hPDC osteo‐layer on top for an additional 24 h fusion period, as illustrated in Figure 3a. Bright‐field imaging showed the formation of a cohesive bilayered structure, with the chondral‐layer (bottom) and hPDC‐derived osteo‐layer (top) maintaining spatial boundaries while integrating at the interface (Figure 3b,c and Figures S9, S10). Histological characterization of iChon+hPDC assembloids revealed dense GAG‐rich matrix staining in the chondral‐layer by SafO/FG and AB/NFR staining, contrasting with a more aligned collagen organization pattern in the hPDC layer visualized by Sirius Red staining under polarized light (Figure 3d). Semiquantitative analysis confirmed significantly higher GAG‐associated staining readouts in the iChon chondral‐layer than in the paired hPDC layer, with the interfacial region (interzone) displaying intermediate GAG‐associated and Sirius Red‐based staining readouts, consistent with a transitional matrix profile (Figure 3e). In contrast, while hAC+hPDC assembloids retained stratified architecture, the hAC chondral‐layer exhibited reduced GAGs deposition compared to iChon based on the SafO/FG and AB/NFR staining, and semiquantitative analysis also revealed a reduced GAG‐associated contrast between hAC and hPDC layers (Figure 3f,g). To substantiate this transition beyond region‐averaged readouts, we performed spatial SafO line‐scan profiling across the junction and defined a SafO‐derived transition thickness (x20−x80, Figure S11). Polarized light imaging further demonstrated less organized collagen alignment in the hPDC layer of hAC+hPDC assembloids, with the interzone showing fragmented matrix continuity compared to the gradual transition observed in iChon+hPDC assembloids. Collectively, these findings show that the modular bioassembly approach generated osteochondral assembloids with zonated matrix deposition, highlighting the intrinsic chondrogenic potential of iChon/hAC and the robust osteogenic capacity of hPDC. The spatial organization and structural integrity of the assembloids validate the utility of spatially guided organoid fusion in engineering complex tissue interfaces.

### 2.4 in vivo Evaluation of Assembloid Constructs in a Full‐Thickness Osteochondral Defect Model in Rats

Following in vitro characterization and zonal bioassembly of iChon+hPDC and hAC+hPDC assembloids, we next assessed their regenerative performance in vivo using a full‐thickness osteochondral defect model with 1.6 mm diameter and 1.6 mm depth in immunocompromised rats. First, the single‐component constructs (hPDC‐only and iChon‐only) were evaluated independently to explore their respective contributions to tissue repair. hPDC‐only constructs were orthotopically implanted into the osteochondral defect and harvested at 8 weeks post‐implantation. Macroscopic examination and 3D reconstruction showed substantial defect filling in the hPDC‐only group (Figure S12a). Quantitative nanoCT analysis further supported a positive contribution of hPDC‐only constructs to subchondral repair, with significantly higher bone volume fraction (BV/TV) in the subchondral defect region compared with defect‐only controls (Figure S12b). However, histological analysis revealed limited restoration of the cartilaginous compartment, as SafO/FG staining showed only partial GAG‐associated staining alongside fibrotic tissue infiltration, whereas defect‐only controls displayed dense fibrous tissue infiltration (Figure S12c). In contrast, iChon‐only constructs were evaluated over a longer period for 16 weeks to assess cartilage stability. Larger iChon‐only constructs were implanted into the same defect model and analyzed via macroscopic and 3D nanoCT imaging. A substantial white tissue fill was observed at the defect site, and both transverse and sagittal hexabrix‐enhanced nanoCT images confirmed the presence of a large, cartilaginous tissue mass (Figure S13a). Histological evaluation by SafO/FG, AB/NFR, and COL II immunohistochemistry staining revealed robust cartilage‐like matrix deposition. Importantly, immunofluorescence staining for Ki67 showed minimal Ki67‐positive cells within the explants, supporting the limited proliferative activity of the implanted iChon‐only constructs over time (Figure S13b). Collectively, these results indicate that hPDC‐only could contribute to subchondral bone formation, while iChon‐only constructs stably maintain a cartilage phenotype after 16 weeks in vivo.

**FIGURE 4:**
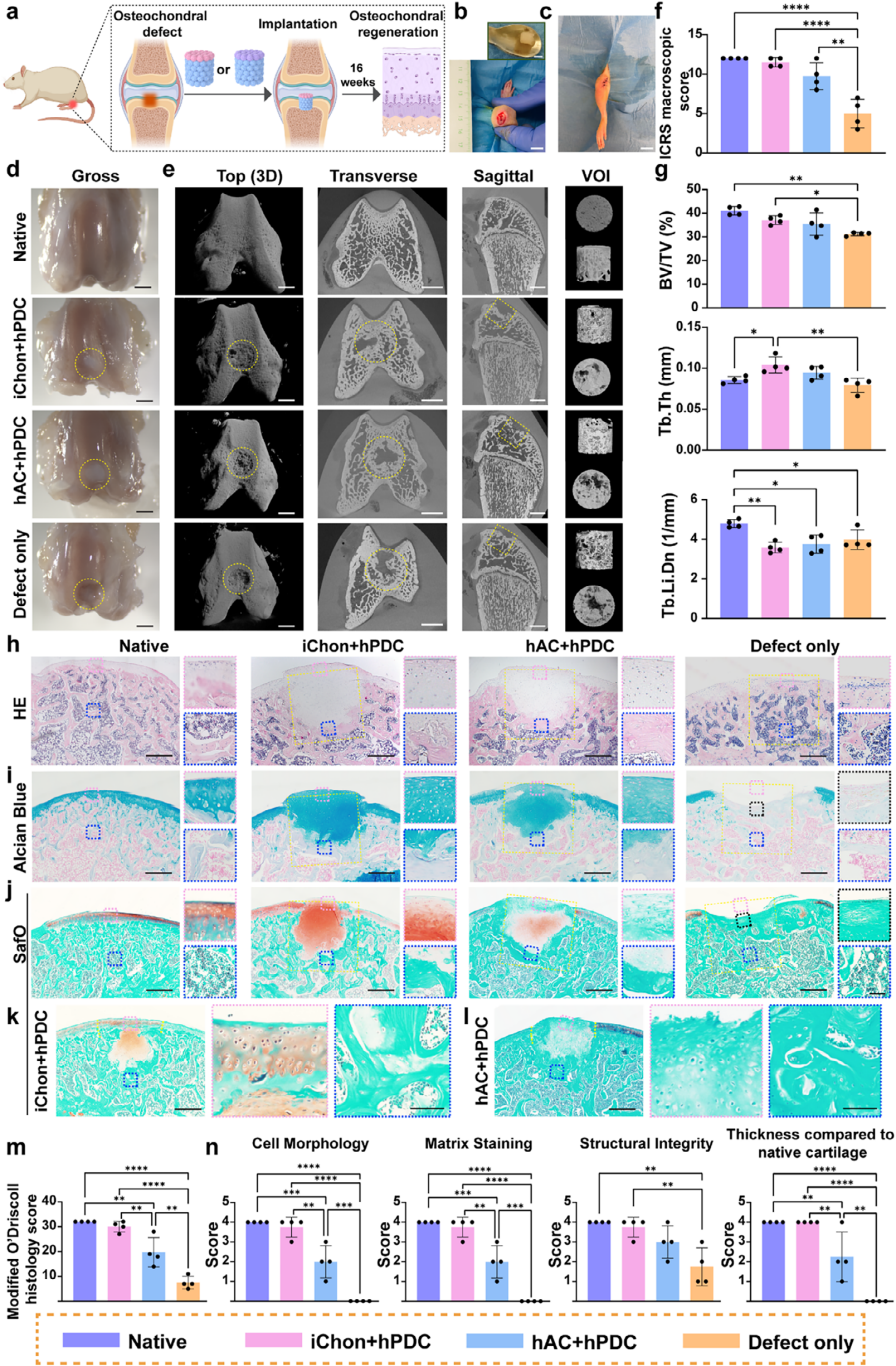
Engineered osteochondral assembloid implants promote structural repair in a rat osteochondral defect model at 16 weeks post‐implantation. (a) Schematic of the rat osteochondral defect model and implantation strategy. (b) Representative images of assembloid implants of iChon+hPDC group and the surgical defect site in a nude rat knee. (c) Post‐implantation closure of the knee joint. (d) Representative images of gross morphology of all conditions. (e) NanoCT images of 3D reconstruction (top), transverse, sagittal views, and volumetric region of interest (VOI, 1.6 mm in diameter × 1.6 mm depth) analysis of explants (native knee as positive control, iChon+hPDC, hAC+hPDC, and defect‐only groups). (Scale bar: 500 µm in panels b–e). (f) International Cartilage Repair Society (ICRS) macroscopic scoring. (g) Quantitative assessment of bone volume fraction (BV/TV), trabecular thickness (Tb.Th), and trabecular linear density (Tb.Li.Dn) based on nanoCT scanning. (h) Hematoxylin and eosin (H&E) staining of regenerated tissues. (i) AB/NFR and (j) SafO/FG staining from representative sections near the defect center. Yellow boxes indicate the defect region. Magenta boxes denote superficial regions adjacent to the articular surface, and blue boxes denote anatomically matched deep repair/subchondral‐side regions within the defect; in the defect‐only group, the black box indicates a slightly deeper location within the defect where tissue is present. (k,l) Additional SafO/FG sections from planes closer to the graft margins for iChon+hPDC and hAC+hPDC groups, respectively. (Scale bar: 500 µm in overview and 100 µm in the zoom‐in images of panels h–l). (m) Modified O'Driscoll histological scoring for cartilage repair quality of all conditions and (n) subcategory analysis of cell morphology, matrix staining, structural integrity, and cartilage thickness compared to native cartilage. For panel g, data presented as mean ± SD; *n* = 4 animals per group, each dot represents one animal; group differences were assessed using one‐way ANOVA followed by Tukey's multiple comparisons test. For panels f and m–n, *n* = 4 animals per group, each dot represents one animal; group differences were assessed using the Kruskal–Wallis test followed by Dunn's multiple comparisons test. ns, not significant; *p < 0.05; **p < 0.01; ***p < 0.001; ****p < 0.0001.

Building on these observations, dual‐layer assembloids (iChon+hPDC or hAC+hPDC) were orthotopically implanted to further evaluate osteochondral repair, with defect‐only included as a negative control (Figure 4a–c). At 16‐week post‐implantation, gross inspection showed more complete defect filling and a smoother surface appearance in the dual‐layer assembloids groups than in defect‐only controls (Figure 4d). No gross adverse events, including joint effusion, infection, necrotic tissue, or macroscopic signs of synovitis, were noted at explantation. NanoCT imaging was further used to assess structural features of repair and mineralized tissue formation within the defect region. The 3D reconstructions, together with transverse and sagittal views, showed that the implanted constructs filled most of the defect volume, although the extent and continuity of mineralized tissue varied across conditions (Figure 4e). In both iChon+hPDC and hAC+hPDC groups, mineralized tissue was present within the defect and extended upward from the defect base. In representative views, partial voids or unmineralized regions were more apparent in hAC+hPDC and defect‐only samples, whereas iChon+hPDC relatively showed a more continuous mineral distribution across the defect region, although small unfilled areas were still observed. Consistent with these observations, volumetric region‐of‐interest (VOI) analysis indicated a more consistent structural outcome within the defect in the iChon+hPDC group. Furthermore, the International Cartilage Repair Society Scoring System (ICRS) was used to evaluate these macroscopic repair results, including defect fill, integration to border zone, and surface appearance (score at 0–4 each) (Table S3). The ICRS macroscopic scores of iChon+hPDC were significantly higher than those of the defect‐only groups and were not significantly different from native tissue (Figure 4f). Quantitative nanoCT analysis of trabecular parameters within the defect VOI was then performed (Figure 4g). BV/TV in the iChon+hPDC group was significantly higher than the defect‐only group (*p* < 0.05) and comparable to native tissue, indicating increased mineralized tissue filling within the subchondral defect region at this time point. The hAC+hPDC group showed an intermediate BV/TV, with no significant differences from either iChon+hPDC or native groups. Importantly, microarchitectural parameters related to trabecular organization/connectivity remained inferior to native tissue across repair groups, consistent with incomplete trabecular maturation/remodeling at 16‐week. Trabecular thickness (Tb.Th) was higher in iChon+hPDC than in native (*p* < 0.05) and defect‐only (*p* < 0.01) but with no significant difference compared to hAC+hPDC. Trabecular linear density (Tb.Li.Dn), measuring the number of trabecular intersections per unit length, was highest in the native group and significantly reduced in both assembloid groups and defect‐only controls. No significant differences were observed in trabecular separation (Tb.Sp) among the groups (Figure S14). Together, the increased BV/TV accompanied by elevated Tb.Th and reduced Tb.Li.Dn indicates a coarser trabecular microarchitecture within the defect VOI at 16‐week, consistent with ongoing remodeling and incomplete defect regeneration relative to native bone [41].

To further investigate the regenerated tissue within the osteochondral defect, histological analysis including H&E, SafO/FG, and AB/NFR staining was carried out. H&E staining revealed that both iChon+hPDC and hAC+hPDC groups exhibited continuous tissue filling of the defect region with good integration into the surrounding host tissue, in contrast to the defect‐only group which displayed incomplete filling and disorganized fibrous tissue (Figure 4h). At higher magnification, the iChon+hPDC group showed a denser and more cartilage‐like cellular organization in the superficial repair region than hAC+hPDC, whereas the defect‐only group lacked comparable cartilage‐like features. AB/NFR staining revealed stronger and more homogeneous Alcian Blue‐positive matrix staining in iChon+hPDC than in hAC+hPDC, while defect‐only samples remained largely negative (Figure 4i). Similarly, SafO/FG staining showed a broader and more intense SafO‐positive repair matrix in iChon+hPDC than in hAC+hPDC, whereas defect‐only samples lacked comparable staining (Figure 4j). Additional SafO/FG sections from planes closer to the graft margins are shown in Figure 4k,l, which reproduce the overall group‐wise pattern while highlighting plane‐to‐plane heterogeneity. Serial stainings spanning the defect center are provided in Figure S15 to further illustrate section‐to‐section heterogeneity across the explant and the representativeness of the selected defect‐center planes. Quantification of SafO‐ and AB‐positive area fractions within the native‐referenced cartilage‐like repair compartment is provided in Figure S17.

In order to semiquantitatively assess cartilage repair, a modified O'Driscoll histological scoring system was further applied across groups (Figure 4m and Table S4). The iChon+hPDC constructs achieved the highest composite scores, which were comparable with native groups, with significantly better cellular morphology, matrix staining, structural integrity and thickness (compared to native cartilage) compared to hAC+hPDC and defect‐only groups (Figure 4n). Given the categorical nature of the thickness item, a direct per‐animal thickness/depth readout is provided in Figure S18. Scores for adjacent tissue integration, surface regularity, chondrocyte clustering and subchondral bone formation were also assessed, further supporting improved repair quality in the iChon+hPDC group (Figure S16). Although hAC+hPDC constructs showed intermediate repair quality, the defect‐only group consistently exhibited the lowest scores across evaluation parameters. Collectively, these histological and quantitative analyses indicate that iChon+hPDC assembloids support osteochondral defect filling with improved cartilage‐like matrix deposition and partial and spatially heterogeneous subchondral maturation/remodeling, while the SafO‐positive region extending into the deep compartment indicates that remodeling remains ongoing at 16‐week.

### 2.5 Protein‐Level Marker Mapping Reveals Articular Surface‐Associated Cartilage Phenotype and Remodeling‐Associated Signatures in the Subchondral Compartment

**FIGURE 5:**
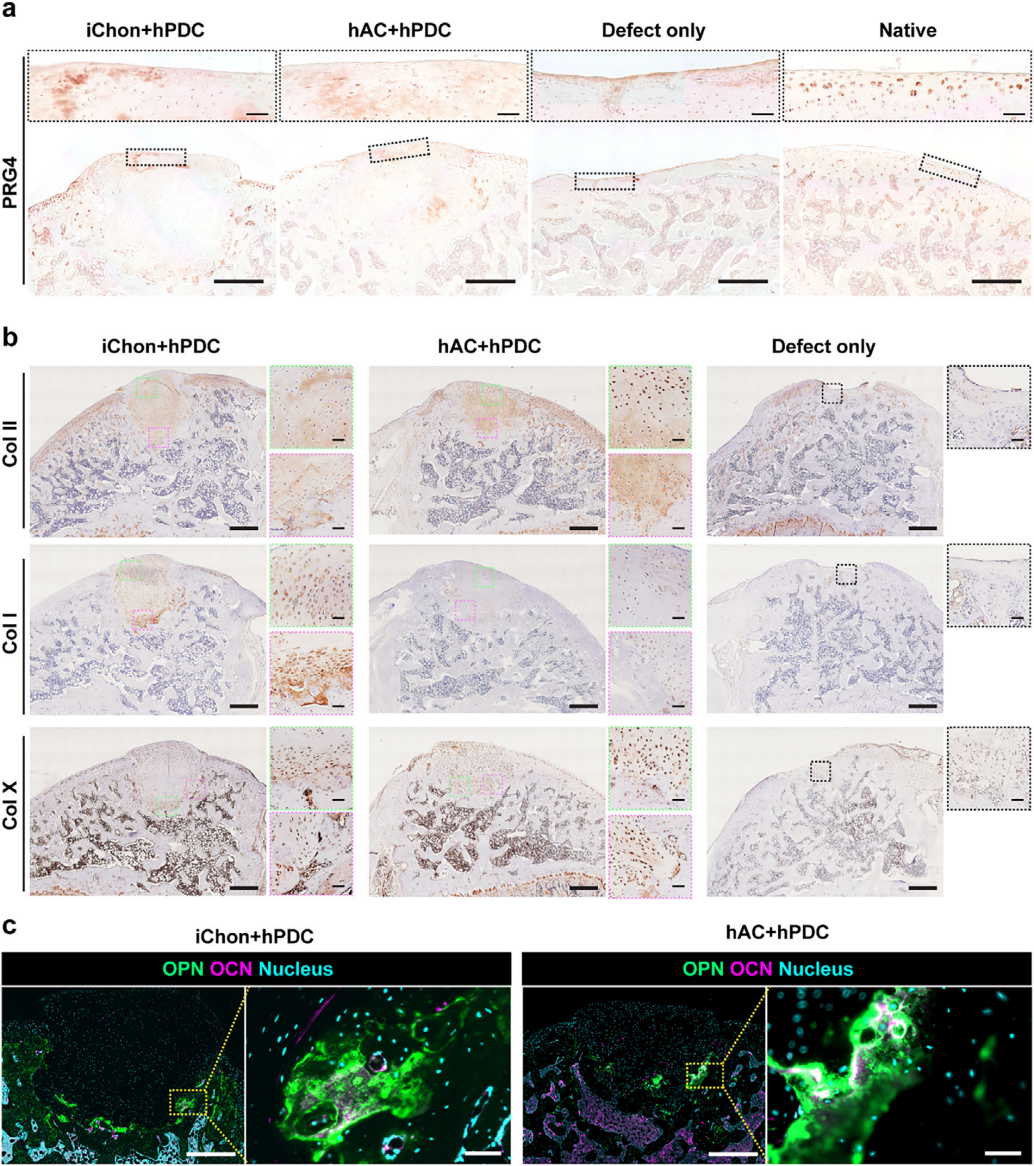
Protein‐level marker mapping supports articular surface‐associated cartilage phenotype and subchondral remodeling after 16 weeks in vivo. (a) Representative PRG4/lubricin immunohistochemistry (IHC) in repaired osteochondral defects across iChon+hPDC, hAC+hPDC, and defect‐only groups, shown alongside native cartilage as reference. Dashed rectangles indicate the articular surface region displayed at higher magnification above each overview image. (b) Representative IHC staining for Col II, Col I, and Col X in explants from iChon+hPDC, hAC+hPDC, and defect‐only groups. Dashed boxes denote regions shown at higher magnification. (c) Immunofluorescence staining for osteopontin (OPN, green), osteocalcin (OCN, magenta), and nuclei (cyan) in repaired defects. Yellow boxes indicate regions shown at higher magnification to visualize bone‐associated matrix signals in the deeper compartment. (Scale bars: 500 µm in overview and 50 µm in the zoom‐in images for all panels).

To further validate tissue identity at the protein level within the repaired osteochondral unit, we performed marker mapping on the 16‐week explants (Figure 5). PRG4/lubricin was assessed by immunohistochemistry as a superficial‐zone marker of the articular surface phenotype [42], with native cartilage included as a reference showing the expected surface‐associated distribution (Figure 5a). Within the repaired defects, PRG4 immunoreactivity was more apparent along the articular surface region in the iChon+hPDC condition, whereas the hAC+hPDC group displayed weaker and less continuous surface staining, and the defect‐only group showed minimal signal within the corresponding region (Figure 5a). In addition, PRG4/COL II immunofluorescence in the iChon+hPDC group visualized a continuous superficial PRG4 deposition overlying a COL II‐rich repair matrix (Figure S19), supporting the presence of a superficial zone–associated phenotype within the regenerated cartilage layer.

In parallel, immunohistochemical mapping of collagen markers provided compositional context across the repaired osteochondral unit (Figure 5b). Collagen II immunoreactivity was detectable within the regenerated cartilage layer in both assembloid groups and appeared more prominent and more continuous in iChon+hPDC compared with hAC+hPDC, whereas defect‐only showed minimal staining in the corresponding region. In contrast, collagen I staining was predominantly associated with the deeper compartment, supporting a compositional contrast between the regenerated cartilage layer and the subchondral region. Collagen X staining was mainly detected in deeper regions of the repaired defects in the assembloid‐treated groups, indicating the presence of hypertrophy‐associated matrix features within the osteochondral repair tissue. Complementary to the immunohistochemistry (IHC) mapping, COL I/COL II immunofluorescence provides an additional spatial view of collagen distribution across groups (Figure S20).

To assess bone‐associated matrix signals within the subchondral compartment, we evaluated osteopontin (OPN) and osteocalcin (OCN) by immunofluorescence (Figure 5c). In both assembloid groups, OPN signal was concentrated in the deeper compartment adjacent to the subchondral region, and high‐magnification views confirmed localized OPN‐positive matrix/structure‐associated staining within the highlighted deep regions (Figure 5c). OCN signal was also detected in the deeper compartment and appeared as discrete regions adjacent to, and partially overlapping with, OPN‐rich areas, consistent with bone‐associated matrix deposition at the repair site. In addition, Tartrate‐resistant acid phosphatase (TRAP) staining revealed TRAP‐positive cells within the deep compartment and along trabecular/subchondral surfaces adjacent to the repaired region (Figure S21), supporting the presence of remodeling‐associated resorptive activity at the 16‐week timepoint. Collectively, these protein‐level markers complement the structural and histological assessments and support cartilage repair accompanied by remodeling‐associated signatures in the subchondral compartment at 16 weeks. Moreover, human mitochondria staining provided human‐specific evidence of donor‐associated signal within the regenerated tissue at 16 weeks (Figure S22), supporting local persistence of human cellular material at the repair site.

### 2.6 Anisotropic Collagen Alignment and Hierarchical Zonation in Engineered Osteochondral Constructs

**FIGURE 6:**
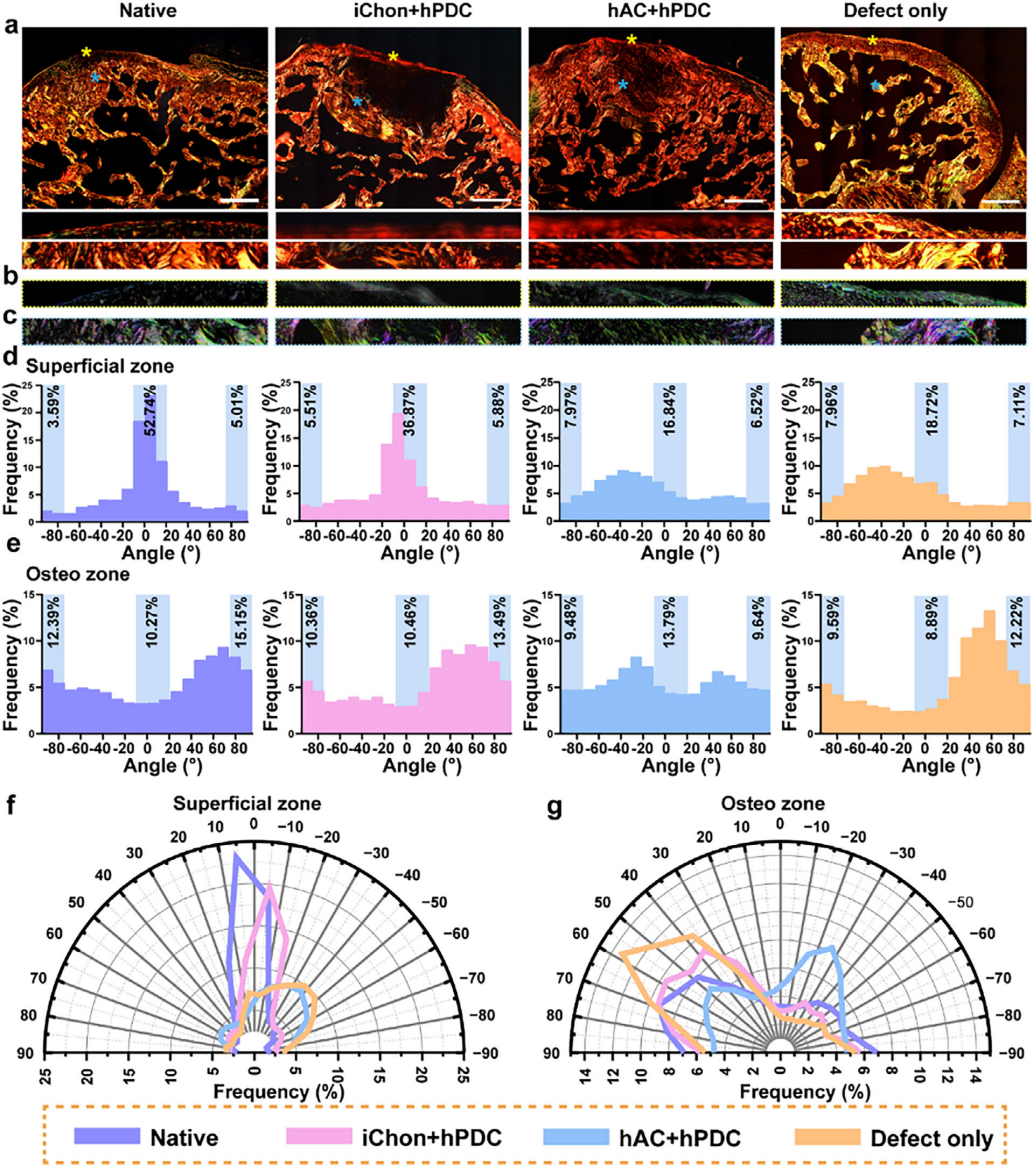
Partial restoration of collagen alignment and osteochondral‐level anisotropy following 16 weeks in vivo regeneration. (a) Sirius Red staining under polarized light microscopy illustrating collagen birefringence and overall fiber organization, birefringence hue is influenced by fiber thickness/packing and orientation (scale bar: 500 µm). (b) Representative ROI definitions for collagen fiber orientation analysis in the superficial cartilage‐like compartment (0–25% depth from the articular surface; superficial ROI) and (c) the deep/subchondral‐side region (osteo zone). (d,e) Comparative fiber orientation distributions relative to native tissue in the superficial zone and osteo zone. (f,g) Polar coordinate mapping of collagen fiber angles in the superficial zone and osteo zone across all groups. For panels f,g, orientation metrics were computed per animal and averaged across ROIs/serial sections prior to plotting.

Given the compartment‐specific matrix signatures observed by marker mapping, we next analyzed collagen fiber architecture using Sirius Red staining under polarizing light microscopy (Figure 6a and Figure S23). In native knee cartilage, collagen fibers exhibit a depth‐dependent anisotropic organization, with fibers aligned parallel to the articular surface in the superficial zone, obliquely in the middle zone and predominantly perpendicular in the deep zone near the subchondral bone [43]. To assess how aspects of this hierarchical organization were recapitulated in the repair tissue, collagen fiber orientation was analyzed separately in an operationally defined superficial cartilage‐like layer at the top of the explants, representing the superficial neocartilage zone (superficial zone) and the deep osteogenic compartment adjacent to the defect base, representing the subchondral region (osteo zone). To further account for depth‐dependent collagen organization, we additionally performed a depth‐resolved analysis across the full cartilage thickness of the cartilage‐like repair compartment by subdividing the region from the articular surface to the cartilage–subchondral transition into four relative‐depth bins (0–25%, 25–50%, 50–75%, 75–100%). Collagen orientation metrics were quantified for each bin using the same Directionality workflow and fixed analysis parameters across all groups (Figure S24). The results show that this architectural hierarchy was partially recapitulated in the iChon+hPDC group, where collagen fibers in the superficial zone displayed partial alignment along the surface‐parallel reference direction and subchondral regions showed a broader orientation profile than the superficial zone (Figure 6b,c). Quantitative orientation analysis of collagen fibers using Directionality plugin in ImageJ demonstrated that in the chondral zone, approximately 52.74% of fibers in native cartilage aligned between −10° to 20°. In comparison, iChon+hPDC assembloids showed 36.87% of fibers within this alignment range, which was higher than hAC+hPDC (16.84%) and defect‐only (17.72%) groups (Figure 6d). In the osteo zones, although collagen fibers in native tissue exhibited less defined orientation due to the more isotropic nature of the subchondral architecture, the iChon+hPDC group displayed the closest orientation profile to the native subchondral region among the repair groups (Figure 6e). Polar coordinate plots further illustrated that collagen fiber distribution in iChon+hPDC assembloids was more concentrated along the dominant orientation axes, whereas hAC+hPDC and defect‐only groups showed broader and more randomized angular distributions (Figure 6f,g). Collectively, these findings indicate an improved yet incomplete restoration of collagen anisotropy in the iChon+hPDC assembloids at 16 weeks, relative to native cartilage, while still outperforming hAC+hPDC and defect‐only controls.

### 2.7 Transcriptomic Profiling Reveals Zonal Contributions of Distinct Cell Populations in Osteochondral Repair

**FIGURE 7:**
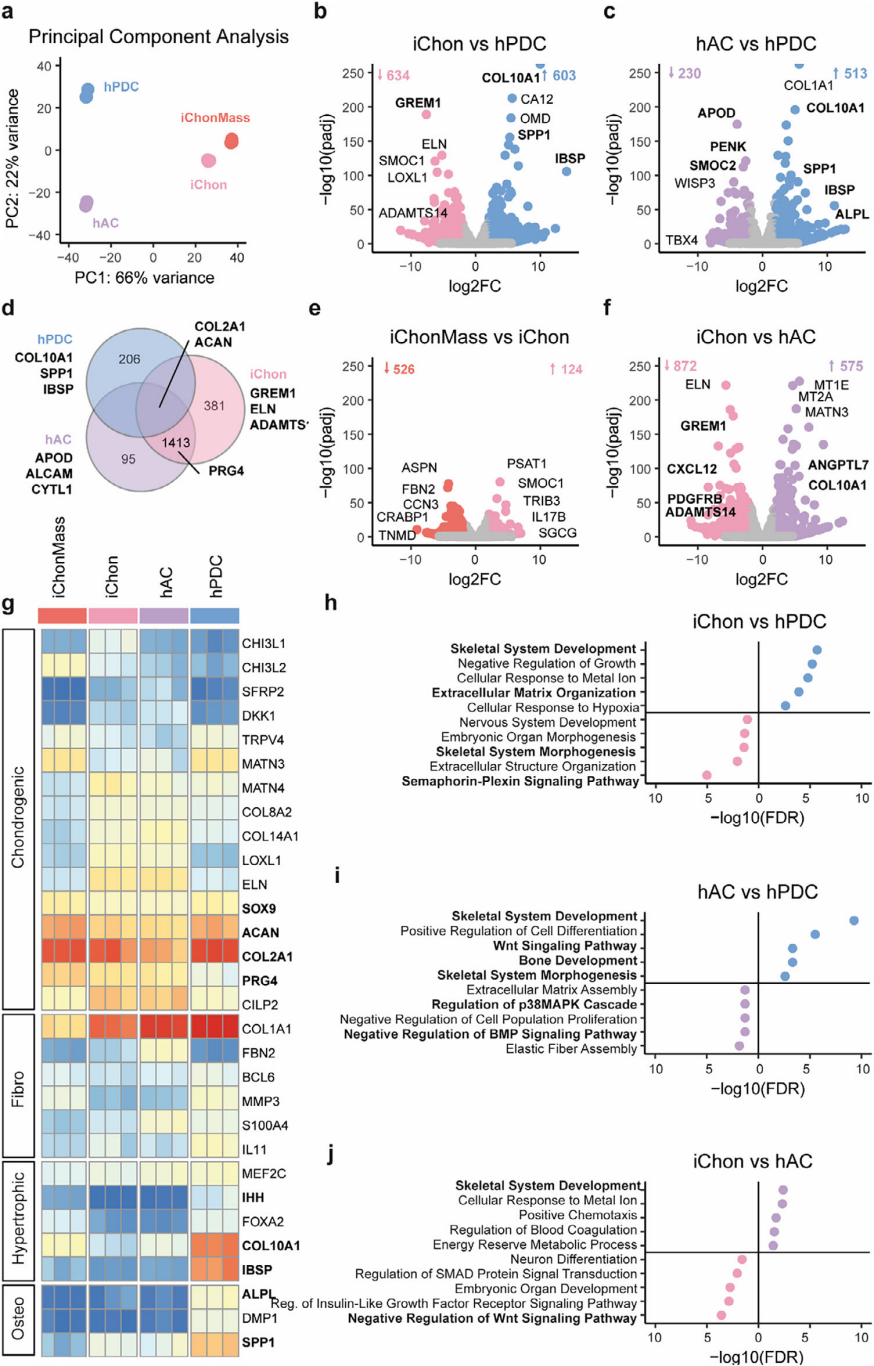
Transcriptomic analysis highlights distinct populations. (a) Principal component analysis (PCA). (b) Differential gene expression analysis (DGE) between day 21 iChon and hPDC organoid populations showing significantly expressed genes using thresholds of false discovery rate (FDR) < 0.05 and |log2FC| > 2. (c) DGE analysis between hAC and hPDC populations. (d) Venn diagram showing the absolute number of population‐specific gene sets. (e) DGE analysis between iChonMass and iChon. (f) DGE analysis between iChon and hAC. (g) Heatmap showing the standardized expression of common cell marker gene sets for chondrocytes (chondro), contaminating fibroblasts (fibro), hypertrophic chondrocytes, and osteogenesis (osteo). (h) Gene ontology biological process enrichment of selected terms between iChon and hPDC, ordered by FDR. (i) Gene ontology biological process enrichment of selected terms between hAC and hPDC. (j) Gene ontology biological process enrichment of selected terms between iChon and hAC. PC, principal component; Log2FC, log2(fold change). Bulk RNA‐seq was performed using *n* = 3 biological replicates per group. Differential expression analysis was performed using DESeq2 with Benjamini–Hochberg adjustment to control the false discovery rate. Partial key marker genes and representative enriched terms are highlighted in bold.

The in vivo outcome is determined by the cellular characteristics of the two zones and their interaction. Hence, transcriptome analysis was performed on day‐21 organoids of each zone as well as the 8‐week iChonMass nodules prior to organoid reaggregation. Principal component analysis revealed distinct clustering of four populations. While both hAC and iChon are categorized as articular cartilage, they still show significant differences (Figure 7a). Differential gene expression (DGE) analysis identified robust upregulation of hypertrophic and early osteogenic genes in hPDC such as *COL10A1*, *SPP1*, and *IBSP* (Figure 7b,c), confirming their identity as transient cartilage progenitors with endochondral ossification potential. The Venn diagram (Figure 7d) showed a shared chondrogenic core among all three organoid types, which likely explains the absence of typical markers such as *ACAN* and *COL2A1* in the DGE analysis. This is further supported by heatmap analysis (Figure 7g), where *COL2A1* and *ACAN* were broadly expressed across all groups. In contrast, *PRG4* expression was restricted to iChon and hAC, confirming their articular cartilage phenotype, while osteogenic markers (e.g., *IBSP*, *SPP1*, *ALPL*) were specifically upregulated in hPDC. Additionally, transcriptional comparison of iChonMass and iChon showed relative similarity (Figure 7e), confirming that reaggregation of iChonMass preserved iPSC‐derived cartilage phenotype. However, comparison between iChon and hAC (Figure 7f) indicated a less mature identity of the iChon populations with higher expression of early progenitor marker genes such as *GREM1*, *CXCL12*, and *PDGFRB*, as well as pro‐collagen N‐peptidase *ADAMTS14*. In contrast, hAC showed slight upregulation of *COL10A1* and *ANGPTL7* (Figure 7f), which are associated with early chondrocyte hypertrophy, but still significantly less expressed than in hPDC (Figure 7c). Finally, gene ontology (GO) enrichment analysis (Figure 7h–j) provided further insight into lineage‐specific functional programs. hPDC were enriched in skeletal development and bone formation pathways (Figure 7h,i), whereas iChon showed enrichment related to nervous system development which has been reported for iPSC‐derived cells [44] and also early embryonic morphogenesis, extracellular matrix remodeling including semaphorin–plexin signaling [45], and negative regulation of Wnt signaling. hAC showed enrichment of pathways linked to a more homeostasis‐associated profile, including p38‐MAPK‐related signatures, together with reduced proliferation‐ and BMP‐related signaling. Together, these results reveal distinct, complementary transcriptional programs across the three populations, supporting their respective functional roles within the zonated osteochondral assembloid and their differential cartilage‐forming versus endochondral/osteogenic potential.

## 3 Discussion

In this study, we present a scaffold‐free, modular approach that assembles iChon and hPDC organoids into spatially organized osteochondral assembloid implants. This strategy enables self‐organized matrix‐mediated continuity between cartilage and bone compartments without reliance on exogenous scaffolds or artificial gradients. in vivo, both iChon+hPDC and hAC+hPDC assembloids supported defect filling and evidence of subchondral repair/remodeling, while iChon+hPDC showed more stable cartilage‐like matrix features in the chondral region with a partial restoration of zone‐dependent collagen orientation compared with controls, although the regenerated tissue remained less organized than native cartilage at this time point. These findings highlight the potential of the zonated assembloid as a scaffold‐free construct‐design strategy for engineering osteochondral architecture in clinically relevant joint defects.

One of the critical considerations in designing such zonated assembloid implants is the selection of cell sources that not only replicate the desired tissue phenotype but also maintain stability after implantation. While hACs have long been considered the gold standard for articular cartilage engineering, their regenerative potential is limited by dedifferentiation during in vitro expansion. For instance, studies have reported that ACs might undergo a loss of *COL2A1* and an increase in *COL1A1* expression within two passages, along with reduced proteoglycan production in standard expansion conditions [46, 47]. In our comparative analysis, hAC organoids initially showed strong chondrogenic features, but iChon organoids displayed more progressive *COL2A1* expression and more homogeneous glycosaminoglycan (GAG)‐rich matrix deposition over time (Figure 1e–g). At the protein level, both iChon and hAC organoids remained COL II‐dominant with low COL I, whereas hPDC organoids showed increased COL I and COL X, supporting the use of iChon/hAC as chondral modules and hPDC as an osteo module (Figure 2). Notably, these iChon in vitro features translated into greater in vivo cartilage stability after 16 weeks (Figure 4h–k and Figure S13), including in the iChon‐only constructs, which preserved hyaline‐like cartilage morphology with minimal Ki67 expression (Figure S13b). These observations are consistent with prior studies reporting improved phenotypic stability of iPSC‐derived tissue models (similar to our iChonMass) for in situ cartilage repair [37, 48]. Unlike large iPSC‐derived tissues, which often lack geometric conformity and thereby limit translational applicability, our strategy dissociates iChonMass into single cells and reaggregates them into smaller, size‐controlled iChon organoids (average area 0.067 mm^2^ at day 21, Figure 1c). This preserves iPSC‐derived chondrogenic features while enabling modular bioassembly and defect‐specific construct customization. This combination of phenotypic stability and geometric adaptability supports the use of iChon as the chondral module within a zonated scaffold‐free osteochondral design. This interpretation should nevertheless be considered in light of the fact that iChon and hAC organoids were maintained in different chondrogenic media (CM1 vs. CM2), selected based on the established culture system for each cell source. Although both media contain the core prochondrogenic growth factors TGF‐β1, BMP‐2, and GDF‐5, media‐dependent effects cannot be excluded and systematic medium cross‐over studies, together with optimized redifferentiation of expanded hAC, may further refine this comparison.

To support the osteo part of the zonated assembloid, we used hPDC organoids, which showed strong upregulation of hypertrophic cartilage and early osteogenic genes such as *COL10A1, RUNX2*, and *OSX* (Figure 1g), reflecting their native potential for endochondral ossification, in line with our previous studies [49, 50]. This lineage priming offers a more direct developmental route to bone‐related repair than complex iPSC‐to‐osteoblast differentiation protocols [51]. Importantly, the hPDC‐only control further supports the biological role of this module: compared with defect‐only controls, hPDC‐only constructs supported repair of the deep/subchondral compartment, while remaining insufficient on their own to restore the full osteochondral unit because the cartilaginous compartment remained poorly reconstituted (Figure S12). Thus, the value of the design lies not in either module alone, but in their coordinated spatial assembly.

In this bilayer design, hPDC organoids are intended to constitute the subchondral (osteo) part, while the transition zone corresponds to the fused boundary between the chondral and osteo modules, where graded matrix remodeling is expected to emerge during maturation [52, 53]. We therefore engineered zonated assembloids through sequential bioassembly of iChon or hAC and hPDC organoids under the same defined assembly conditions. The iChon+hPDC assembloids showed dense GAG‐associated matrix in the chondral zone (iChon zone) and collagen‐associated organization in the osteo zone (hPDC zone). The interfacial zone (interzone) showed intermediate matrix characteristics (Figure 3e). Such graded organization emerged without the use of exogenous materials or artificial gradients, suggesting that cell‐driven morphogenesis is sufficient to initiate interface continuity, recalling embryonic joint developmental processes [54, 55]. Given that abrupt cartilage–bone discontinuities can concentrate interfacial stresses, this interdigitated transition zone may support more gradual load transfer across the junction [56, 57]. In comparison, hAC+hPDC showed diminished interfacial coherence and disorganized collagen distribution (Figure 3f), possibly reflecting differences in matrix organization [58]. Although fusion behavior may also contribute to subsequent assembloid maturation [50, 59], both groups were assembled within the same defined sequential workflow. Within this context, the observed structural differences are more plausibly linked to module‐specific biological properties and matrix organization. These structural differences prompted further evaluation of whether such intrinsic matrix organization could translate into improved repair in vivo.

To determine whether this intrinsic matrix organization translated into improved repair in vivo, we next examined the 16‐week outcomes in a full‐thickness osteochondral defect model (Figure 4). At 16 weeks post‐implantation, the iChon+hPDC group demonstrated substantial restoration of the cartilage compartment, whereas the deep compartment showed spatially heterogeneous subchondral repair/remodeling across the defect volume and had not yet reached a fully mature native‐like state. Histological analyses revealed continuous defect filling with hyaline‐like matrix and contiguous integration with host tissue (Figure 4h–k). The SafO‐positive zone extended deeper than native cartilage, particularly in the defect‐center planes, likely reflecting delayed but ongoing remodeling of the subchondral compartment, as has been observed in other studies [60, 61]. Notably, additional planes closer to the graft margins showed a less SafO‐positive and more remodeled deep compartment, supporting the interpretation that deep‐compartment maturation remained spatially heterogeneous rather than absent (Figure S15). Quantitative nanoCT analysis confirmed improved bone volume fraction relative to defect‐only groups (Figure 4g), and modified O'Driscoll scoring confirmed improved histological quality (Figure 4m,n), an established preclinical histological metric of cartilage repair quality [62]. In this context, the additional marker‐based phenotyping (Figure 5 and Figures S19–S21) provides complementary information at the repair interface, with remodeling‐associated readouts (e.g., COL X together with OPN/OCN and TRAP) supporting the notion that remodeling activity remains ongoing in the deep compartment at 16 weeks. While this ongoing maturation suggests that the present findings reflect a mid‐term snapshot of the regenerative process, the substantial deposition of proteoglycan‐rich matrix may provide a matrix basis relevant to later mechanical function [63]. Nevertheless, longer‐term follow‐up incorporating direct biomechanical characterization will be required to confirm durable maintenance of the hyaline‐like cartilage phenotype and the progression of the deep compartment toward more mature subchondral architecture.

Furthermore, structural analysis of our assembloids revealed that the iChon+hPDC partially reestablished the key features of native collagen anisotropy with partial alignment along the surface‐parallel reference direction in the superficial zone and a broader orientation distribution in deep/subchondral region (Figure 6). This hierarchical matrix architecture is essential for resisting multidirectional mechanical loads and maintaining tissue durability, with depth‐dependent fiber realignment known to occur during postnatal development in response to mechanical stimuli [63, 64, 65]. Compared with hAC+hPDC or defect‐only groups, only the iChon+hPDC assembloids demonstrated a more physiologically relevant anisotropy, approaching but not yet fully matching the native organization (Figure 6), suggesting that both the cellular source and the modular bioassembly process contribute to the reestablishment of this anisotropic architecture [66, 67]. Recent studies have sought to reproduce this architecture using biofabrication techniques such as melt electrowriting [68], magnetic field for the alignment of microgels [69], and filamented light biofabrication [70] to obtain aligned or pre‐aligned collagen structure in cartilage tissue engineering. Moreover, recent efforts using plastic compression to control fiber orientation in cartilage organoids further support the critical role of collagen anisotropy in functional tissue reconstruction [71]. In contrast, our results suggest that intrinsic cell behavior, when spatially assembled, can support the emergence of a partially anisotropic architecture in vivo under the biomechanical environment of the knee joint.

To gain insight into the biological basis of the distinct post‐implantation repair trajectories, we performed transcriptomic analysis of the pre‐implantation organoids (Figure 7). Relative to hAC, iChon was associated with higher *GREM1* expression together with enrichment of regulatory programs related to negative regulation of Wnt signaling and SMAD‐associated signaling. These features are consistent with a chondral state less permissive to hypertrophic or endochondral progression and more supportive of stable cartilage properties [72, 73, 74]. In parallel, the higher expression of *ADAMTS14* in iChon, together with enrichment of insulin‐like growth factor receptor signaling, suggests greater competence for extracellular‐matrix synthesis and remodeling [75, 76]. Such pre‐implantation differences in the chondral compartment may have rendered the iChon‐containing assembloids more permissive to organized cartilage‐like repair after implantation, thereby contributing to the distinct repair trajectory observed in vivo. This interpretation is also in line with our previous observation that less mature cartilage implants may better maintain their phenotype in the post‐implantation environment than adult‐derived articular chondrocytes [77]. In parallel, hPDC organoids were characterized by upregulated *COL10A1*, *IBSP*, and *SPP1*, together with enrichment in skeletal system development and bone development, consistent with their transient hypertrophic profile linked to endochondral ossification [78, 79]. Given the zonated architecture of the osteochondral unit, differences in the properties of the cartilage compartment may also secondarily influence remodeling of the underlying hPDC compartment through cartilage–bone crosstalk across the osteochondral junction [56, 57, 80]. However, this remains a biologically grounded hypothesis rather than direct proof of causality. Moreover, full maturation of the osteochondral unit, including stable articular cartilage, tidemark formation, and more complete subchondral restoration, will likely require a longer observation period.

While this study demonstrates a scaffold‐free strategy for osteochondral repair, its broader significance lies not only in its repair outcome but also in its construct‐design principle, in which lineage‐specific organoids act as programmable living building blocks for a zonated biological architecture. Clinically, cartilage/osteochondral defects are commonly treated using marrow‐stimulation procedures (e.g., microfracture), cell‐based cartilage repair (ACI/MACI), or osteochondral auto/allograft transplantation, each associated with recognized constraints (e.g., fibrocartilaginous repair and/or limited restoration of the osteochondral unit, as well as tissue availability, donor‐site morbidity, and integration challenges) [81, 82, 83]. Against this clinical backdrop, our scaffold‐free assembloid strategy is designed to support coordinated regeneration of both chondral and subchondral compartments within a single implant. However, direct benchmarking against these procedures will require future studies incorporating clinically matched comparators and functional endpoints (e.g., gait analysis and/or hindlimb weight‐bearing asymmetry), which were not included in the present study and therefore limit direct structure–function correlations in this model. Beyond efficacy benchmarking, translation of iPSC‐containing implants will also require addressing manufacturing and safety considerations [84, 85]. In this context, the present dual‐cell configuration may offer a pragmatic de‐risking advantage: by using primary cells for the high‐volume bone phase, the total “iPSC load” within the construct is reduced, which may help limit part of the safety and manufacturing burden associated with large‐scale pluripotent cell integration, including concerns related to genomic instability or unintended lineage divergence [86].

Future studies may also benefit from integration with biofabrication strategies for spatially positioning of organoids such as aspiration assisted bioprinting [87, 88], laser assisted bioprinting [89], and extrusion bioprinting could facilitate automation/standardization of such implants. In addition, the application of dynamic mechanical conditioning during in vitro maturation may further enhance matrix anisotropy and improve long‐term mechanical integration [90]. At the same time, the use of immunocompromised animals represents a limitation, as it does not fully reflect the role of host immunity in joint repair. Previous studies have shown that immune cells play a critical role in tissue healing and regeneration, and their absence can lead to altered repair outcomes [91, 92]. Hence, future validation in immunocompetent models will be essential. Collectively, this work contributes to the development of osteochondral assembloid implants with increased complexity yet programmed design specifications. These implants capture key aspects of native regenerative and developmental principles and provide a promising foundation for next‐generation osteochondral repair strategies.

## 4 Conclusion

This study demonstrates that scaffold‐free modular assembly of lineage‐specific organoids enables the generation of structurally and biologically zonated osteochondral constructs. By integrating iChon organoids with hPDC organoids in a spatially guided manner, we obtained implants that supported hyaline‐like cartilage formation together with ongoing, spatially heterogeneous subchondral remodeling in a full‐thickness osteochondral defect model. The resulting assembloids recapitulated several key compositional and architectural features of the osteochondral unit, including partial restoration of collagen anisotropy, while maintaining in vivo stability of the cartilage‐forming module. This zonated assembloid platform holds translational promise for personalized osteochondral therapies and offers a developmentally inspired blueprint for engineering complex skeletal tissue implants.

## 5 Experimental Section

### 5.1 Ethics Statement

The study involving human participants was reviewed and approved by the Ethical Committee for Human Medical Research at KU Leuven (Approval No. ML7861). All patients provided written informed consent. Animal experiments were approved by the Animal Ethics Committee of KU Leuven (P36/2016 ECD and P059/2020) and conducted in compliance with the housing standards of Animalium Leuven (KU Leuven).

### 5.2 Generation of iChonMass

The human‐induced pluripotent stem cell (hiPSC) line BIONi010‐C was obtained from the European Bank for induced Pluripotent Stem Cells (EBiSC) and cultured under previously described protocol [93], with minor adaptations. Initially, hiPSC were maintained on mitomycin C‐treated SNL feeder layers in StemFit Basic 04 medium (Ajinomoto, SF041‐001) under standard humidified conditions (37°C, 5% CO_2_). Medium was changed daily, and cells were passaged weekly. For feeder removal and transfer to feeder‐free culture, colonies were first gently digested with CTK solution (0.25% trypsin, 1 mg/mL collagenase IV, 20% knockout serum replacement in 0.1 m CaCl_2_ solution) to detach feeder cells while minimizing disturbance to the iPSC colonies. After thorough phosphate‐buffered saline (PBS) washing to eliminate residual feeder cells, colonies were manually fragmented into small clumps and transferred onto Matrigel (Becton Dickinson)‐coated 6‐well plates (Nunc Thermo Scientific). Cells were then cultured in Essential 8 medium (Gibco, A1517001) for two weeks to establish a feeder‐free monolayer prior to differentiation.

Next, feeder‐free iPSC were subjected to mesoderm induction following a two‐step protocol. Initially, cells were cultured in Essential 6 medium supplemented with 8 µm CHIR99021 (Axon Medchem, Axon1386), 20 ng/mL FGF‐2 (Peprotech, 100‐18C), and penicillin/streptomycin (Pen/Strep, Gibco, 15140122) for 36 h. This was followed by 36 h of treatment with Essential 6 medium containing 1 µm retinoic acid (RA; Sigma‐Aldrich, R2625) and 8 ng/mL FGF‐2. Subsequently, cells were differentiated toward the chondrogenic lineage using chondrogenic medium (CM), consisting of high glucose Dulbecco's Modified Eagle Medium (DMEM) (Gibco, 11995065) supplemented with 1% fetal bovine serum (FBS; Hyclone), 1% L‐glutamine, 1% nonessential amino acids (Gibco, 11140050), 1% ITS‐X (Gibco, 51500056), 50 µg/mL ascorbic acid, 0.1 mm 2‐mercaptoethanol, 10 ng/mL each of FGF‐2, TGF‐β1 (PeproTech, 100‐21C), BMP‐2 (PeproTech, 120‐02), GDF‐5 (PeproTech, 120‐01), and 50 U/mL Pen/Strep. Chondrogenic induction continued until day 14, with medium refreshed every two to three days. At day 14, emerging cartilage‐like nodules were manually collected and transferred to nonadherent suspension culture in CM medium without FGF‐2 (CM1) with an additional six weeks culture. And the medium was changed twice weekly, to promote 3D maturation. By the end of the culture period (week 8), mature iPSC‐derived chondrocytes macromass (iChonMass) constructs with diameters of approximately 2–3 mm were obtained.

### 5.3 iChonMass Chondrogenic Phenotype Stability by Ectopic Implantation

To validate the long‐term stability of the chondrogenic phenotype of iChonMass, ectopic implantation was performed in NMRI^nu/nu^ nude mice (Jackson laboratory; *n* = 4; 8 week‐old females). Prior to transplantation, iChonMasses were gently collected and washed twice in sterile 1× DPBS at room temperature. The washed iChonMasses were then implanted into the subcutaneous tissue pockets created on the dorsum of anesthetized mice. After 8 weeks in vivo, animals were euthanized, and explants were harvested for analysis. The explants were fixed in 4% paraformaldehyde (PFA) for subsequent nanoCT scan and histological analysis.

### 5.4 iChon Single Cells Dissociation of iChonMass for Organoid Formation

To generate single cell suspension from iChonMass nodules which obtained from section 5.2 , a two‐step enzymatic digestion protocol was employed. Briefly, approximately 40 nodules were collected and initially incubated with 2 mg/mL Pronase (Roche, 10165921001) dissolved in PBS supplemented with 1% Pen/Strep at 37°C under gentle agitation. Digestion time was adjusted according to nodule size, ranging from 20 min for small nodules to 30 min for larger ones. After Pronase treatment, nodules were washed and subjected to further digestion with 1.5 mg/mL Collagenase B (Roche, 11088815001) dissolved in DMEM/F12 medium (Gibco) containing 1% Pen/Strep. Collagenase digestion was carried out at 37°C with constant rotation for up to 4 h, with regular monitoring to ensure optimal tissue dissociation. Following enzymatic digestion, the cell suspensions were filtered through a sterile 70 µm cell strainer (Fisherbrand, 22363548) to remove undigested debris, and then centrifuged for 10 min at room temperature. After centrifugation, the supernatant was carefully aspirated, and the resulting cell pellets were resuspended in CM1 medium. The cell viability and total cell number were assessed using a hemocytometer or automated cell counter. On average, approximately 200 000 viable cells were recovered per nodule, suitable for subsequent organoid formation seeding.

### 5.5 Human Periosteum‐Derived Cells and Articular Chondrocytes Isolation and Culture

hPDC were isolated from periosteal biopsies obtained from six healthy donors (*n* = 6; age range 20–32 years; 5 male, 1 female; coded donor IDs: HP297, HP307, HP328, HP355, HP390, HP426) and pooled to create a single mixed donor cell pool, as previously described [94]. Briefly, periosteal tissue was enzymatically digested to obtain single‐cell suspensions, which were subsequently expanded in DMEM (Gibco) supplemented with 10% FBS (biowest) and 1% antibiotic–antimycotic solution (100 U/mL penicillin, 100 µg/mL streptomycin, and 0.25 µg/mL amphotericin B; Gibco, 15240062). Cells were cultured under 37°C, 5% CO_2_, 95% humidity, with medium changes every 2–3 days. At 80–90% confluence, cells were detached using 0.25% TrypLE Express (Life Technologies) and further expanded for 6 passages.

Healthy hAC (Lonza, CC‐2550) were commercially purchased and expanded at 37°C, 5% CO_2_, 95% humidity. Cells were cultured according to the manufacturer's guidelines. Medium changes were performed every 2–3 days, and cells were passaged at 80–90% confluence using 0.25% TrypLE Express. Passage 3 were used for the organoid formation and subsequent in vivo studies.

### 5.6 Formation of Organoid From iChon, hAC, and hPDC

A nonadherent microwell platform (AggreWell800, STEMCELL Technologies Inc) was prepared by coating with anti‐adherence rinsing solution (STEMCELL Technologies Inc) to prevent cell attachment, followed by washing with basal DMEM medium prior to cell seeding. The harvested iChon, hAC, and hPDC single cells were respectively seeded into the AggreWell at 300 000 cells per 2 mL differentiation medium to promote self‐aggregation. Half of the medium was changed twice per week for a period of 21 days. Specifically, hPDC and hAC were respectively cultured in chemically defined chondrogenic medium (CM2), composed of low‐glucose DMEM (Gibco) supplemented with 1% antibiotic–antimycotic solution, 1 mm L‐Ascorbic acid 2‐phosphate sesquimagnesium salt hydrate (Sigma‐Aldrich, A8960), 100 nm dexamethasone (Sigma‐Aldrich, D4902), 40 µg/mL proline (Sigma‐Aldrich, P5607), 20 µm of Rho‐kinase inhibitor Y27632 (Axon Medchem, 1683), ITS + Premix containing 6.25 µg/mL insulin, transferrin and selenious acid (Corning, 354352), 100 ng/mL GDF‐5, 100 ng/mL BMP‐2 (InductOs), 10 ng/mL TGF‐β1, 1 ng/mL BMP‐6 (PeproTech, 120‐06), and 0.2 ng/mL FGF‐2. iChon organoids were cultured in CM1 medium. All organoids were cultured at 37°C, 5% CO_2_ and 95% humidity.

### 5.7 Zonated Osteochondral Assembloids Formation

Macro‐agarose wells with 2 mm diameter were prepared by pouring 3 mL of molten 3% agarose into each well of a 24 well plate, immediately inserting 3D‐printed PDMS cylindrical molds into the molten agarose. After solidification at room temperature, the molds were carefully pulled out to create standardized agarose wells. Each well was then flushed with 1 mL CM1 media to permeabilize the agarose mold, followed by UV sterilization for 30 min. For the formation of dual constructs, day 21 iChon organoids were gently flushed out from the Aggrewell platform, concentrated, and seeded into the macro‐agarose wells and the chondral and osteo parts were assembled using a volumetric loading ratio of 1:5. The same volumetric loading ratio was applied to both iChon+hPDC and hAC+hPDC assembloids. Organoids were allowed to sediment naturally for 30 min at room temperature. After that, 2 mL of CM1 media was added to each well, and constructs were cultured at 37°C, 5% CO_2_ and 95% humidity for an additional 24 h to promote initial fusion and stabilization of the chondral layer. Afterward, hPDC organoids were seeded atop the iChon organoid layer, followed by another 24 h of culture at 37°C, 5% CO_2_ and 95% humidity in CM1 medium, thereby generating the zonated iChon+hPDC assembloids. In parallel, hAC organoids were similarly combined with hPDC organoids to form hAC+hPDC assembloids, following identical procedures. Successful fusion was operationally defined by interface continuity on bright‐field inspection and stability during standardized retrieval/transfer (no visible delamination). Additionally, constructs composed solely of iChon or hPDC organoids were prepared for control experiments (iChon only or hPDC only).

### 5.8 Gene Expression Analysis

Organoids derived from iChon, hAC, and hPDC were harvested at day 7, 14, and 21. For each replicate, approximately 300 organoids from one AggreWell plate were pooled. For iChonMass samples, mechanical disruption was performed prior to RNA extraction using Precellys lysing kits (VWR, 432‐0141) to ensure complete homogenization of the dense tissue. Total RNA was extracted for all samples using the RNeasy Mini Kit (Qiagen, 74134) following the manufacturer's protocol. RNA concentration and purity were assessed via NanoDrop 2000 spectrophotometer (Thermo Fisher Scientific), with acceptable thresholds defined as A260/A280 ≈2.0 (protein purity) and A260/A230 between 2.0–2.2 (salt/organic contaminant purity). cDNA was synthesized from 500 ng of total RNA using the PrimeScript RT reagent kit (Takara, PR037A). reverse transcription quantitative PCR (RT‐qPCR) was performed on a Rotor‐Gene 6000 system (Qiagen) with SYBR Green fluorescence detection (Life Technologies). Thermocycling conditions included an initial denaturation at 95°C for 10 min, followed by 40 cycles of denaturation at 95°C for 10 s, annealing at 62°C for 15 s, and elongation at 72°C for 20 s. Melt curve analysis was performed after amplification to confirm primer specificity, and all reactions were run in technical replicates. Gene expression levels were normalized to *β‐ACTIN (β‐ACT*), and relative expression was calculated using the 2^−ΔΔC^ *^t^* method [95]. Primer sequences are listed in Table S2.

### 5.9 Bulk RNA Sequencing Analysis

D21 iChon, hAC, and hPDC organoids, as well as iChonMass samples, were collected to generate biological replicates (*n* = 3 per group). For dense iChonMass tissues, mechanical disruption was performed using Precellys lysing kits prior to RNA isolation. Total RNA was extracted using the RNeasy Mini Kit and quantified by NanoDrop analysis. Only samples with acceptable A260/A280 and A260/A230 ratios were processed further. RNA sequencing libraries were prepared using the Illumina TruSeq Stranded mRNA Library Preparation Kit according to the manufacturer's protocol. Briefly, RNA was denatured at 65°C, fragmented, and reverse‐transcribed into cDNA with indexed adapters for multiplexed sequencing. Sequencing was conducted by the Genomics Core Leuven on an Illumina HiSeq4000 platform, generating single‐end 75 bp reads. Raw sequencing reads were quality‐checked with FastQC v0.11.7. Adapter sequences were trimmed using Trimmomatic v0.39 [96]. Splice‐aware alignment to the human reference genome (hg38) and transcriptome was performed using HISAT2 [97] with default settings. Gene‐level quantification of uniquely mapped reads was conducted with Feature Counts from subread package [98], using strand‐specificity (‐s 1) and gene symbols for annotation (‐g gene_name). Differential expression analysis was performed using DESeq2 [99] in R, filtering low‐expression genes (mean counts < 10) and excluding mitochondrial/ribosomal genes. Adjusted *p*‐values were calculated using the Benjamini–Hochberg procedure to control the false discovery rate (FDR < 0.05) [100]. Gene ontology analysis was performed by utilizing the EnrichR package in R.

### 5.10 Osteochondral Defect Implantation in Rats

Orthotopic implantation experiments were conducted in female athymic nude rats (ENVIGO, Hsd: RH‐Foxn1^rnu^, 10 weeks old at surgery). A full‐thickness osteochondral defect (1.6 mm diameter × approximately 1.6 mm depth) was surgically created in the femoral trochlear groove of one knee per animal following an established procedure [101]. The experiment was performed in two phases and all rats were randomized into groups with 4 rats per condition. First, the respective layers were evaluated individually where hPDC‐only constructs were implanted and maintained in vivo for 8 weeks, whereas iChon‐only constructs were implanted and maintained for 16 weeks, to evaluate their respective behaviors within the joint environment.

A second implantation phase was carried out to assess the therapeutic efficacy of zonated assembloid constructs over a 16‐week in vivo period. Experimental groups consisted of iChon+hPDC constructs, hAC+hPDC constructs, and an empty defect‐only as a control group. The assembloids were implanted using a press‐fit technique, where the construct diameter matched the defect size to ensure primary mechanical stability. Following insertion, the surgical site was maintained in a relatively dry state for approximately 5 min to allow for initial stabilization and to prevent flotation through endogenous fibrin adhesion. The constructs were carefully placed into the defects, allowed to stabilize for several minutes, and the patella was repositioned before closing the surgical site in layers. After the operation, rats were allowed unrestricted cage activity and were administered intramuscular penicillin to prevent infection. Animals were maintained under standard housing conditions with access to food and water ad libitum. At 16‐week post‐implantation, animals were euthanized, and the operated knees were harvested for histological and NanoCT analyses. Contralateral nonoperated knees served as healthy controls (Native groups).

### 5.11 NanoCT Imaging and Quantification

Explanted samples, including ectopic (subcutaneous iChonMass, 8 weeks) and orthotopic (knee defects), were fixed in 4% PFA overnight at 4°C. Samples were then immersed in 20% Hexabrix (Guerbet, France) staining solution overnight at 4°C to enhance cartilage contrast for imaging. All samples were scanned using a Phoenix NanoTom M system (GE Measurement and Control Solutions) at 5 µm voxel resolution. Orthotopic explants were scanned using the following parameters: 80 kV voltage, 131 µA current, 0.2 mm aluminum filter, and 500 ms exposure time per frame across 1800 projections. Ectopic samples were scanned at 60 kV voltage, 170 µA current, 0.1 mm aluminum filter, and 500 ms exposure time per frame across 1800 projections. Image reconstruction was performed using Datos X software (Bruker‐µCT, BE), followed by spatial alignment of samples to standardize analysis of subchondral bone and cartilage regions. For orthotopic defects, a cylindrical VOI with 1.6 mm diameter × 1.6 mm height was defined, corresponding to the original defect size. Ectopic constructs were analyzed in their entirety. Quantitative morphometric analyses, including bone volume fraction (BV/TV), Tb.Th, Tb.Sp, and Tb.Li.Dn, were performed using CTAn software (Bruker‐µCT). Contralateral noninjured knees (orthotopic groups) served as healthy controls. 3D visualization of mineralized and Hexabrix‐stained cartilage regions was generated using CTVox software (Bruker‐µCT).

### 5.12 Histology and Immunostaining

Subcutaneous explants and knee joint samples were fixed in 4% PFA at 4°C overnight. Subcutaneous tissues were subsequently decalcified in 0.5 m ethylenediaminetetraacetic acid (EDTA)/PBS (pH 7.5) for 10 days at 4°C, while knee joints were decalcified for 1 month. After decalcification, all tissues were embedded in paraffin and sectioned into 10‐µm‐thick slices using a microtome.

For histological evaluation, sections were deparaffinized in HistoClear (National Diagnostics, HS‐200) and rehydrated through a graded ethanol series (100%, 95%, 70%, 50%), followed by routine staining procedures as previously described [102]. H&E staining was performed to assess general tissue architecture. Proteoglycan‐rich matrix distribution was evaluated using Safranin O/Fast Green staining (0.25% Safranin O in aqueous solution; SafO/FG) with hematoxylin counterstaining. Acidic proteoglycans were visualized by Alcian Blue/Nuclear Fast Red staining (0.5% Alcian Blue in 1 m HCl) followed by Nuclear Fast Red counterstaining (AB/NFR). TRAP staining was performed using the Leukocyte Acid Phosphatase (TRAP) Kit (Sigma‐Aldrich, 387A) according to the manufacturer's instructions.

For knee joint analysis, serial sections spanning the entire defect region were collected, and representative sections from the defect center together with adjacent planes were selected to ensure evaluation at comparable anatomical levels across samples. For Safranin O/Fast Green and Alcian Blue/Nuclear Fast Red histomorphometry, a predefined native‐referenced cartilage‐like repair compartment was selected within the defect region based on the adjacent native cartilage plane, excluding deeper staining extension into the deep compartment. Positive staining area fraction (%) was quantified in Fiji/ImageJ and averaged across three serial sections per animal.

For IHC, deparaffinized sections underwent antigen retrieval in Tris‐EDTA buffer (10 mm Tris base, 1 mm EDTA, pH 9.0) at 95–98°C for 20 min, followed by cooling to room temperature. Endogenous peroxidase activity was quenched with 3% hydrogen peroxide for 10 min, and nonspecific binding was blocked with 2.5% bovine serum albumin (BSA) containing 0.3% Triton X‐100 in PBS for 30 min at room temperature. Primary antibodies were diluted in blocking buffer and incubated overnight at 4°C. For the in vivo marker mapping, IHC was performed for PRG4/lubricin (Abcam, ab28484; 1:300), COL I (Thermo Fisher, MA1‐141; 1:200), COL II (Merck, AB761; 1:50), and COL X (Cosmo Bio, LSL‐LB‐0092). Negative control sections were processed in parallel using the same procedure but without primary antibody. Sections were then incubated for 1 h at room temperature with HRP‐conjugated anti‐rabbit IgG (Jackson ImmunoResearch, 111‐035‐003; 1:500) or HRP‐conjugated anti‐mouse IgG (Jackson ImmunoResearch, 115‐035‐146; 1:500), followed by Diaminobenzidine (DAB) development and hematoxylin counterstaining. Sections were dehydrated through graded ethanol, cleared in HistoClear, and mounted with Pertex medium.

For immunofluorescence (IF), deparaffinized sections underwent antigen retrieval and blocking as described above, followed by overnight incubation at 4°C with primary antibodies diluted in blocking buffer. Depending on the experiment, IF staining was performed using antibodies against Ki67 (Thermo Fisher, MA5‐14520; 1:50), COL I (Thermo Fisher, MA1‐141; 1:200), COL II (Merck, AB761; 1:50), COL X (Cosmo Bio, LSL‐LB‐0092; 1:200), PRG4/lubricin (Abcam, ab28484; 1:300), osteopontin/OPN (R&D Systems, AF1433; 1:200), osteocalcin/OCN (Abcam, ab198228; 1:100), and human mitochondria/hMito (NeoBiotechnologies, RBM6‐2860‐1; 1:100). After washing, sections were incubated with species‐specific Alexa Fluor‐conjugated secondary antibodies, including Goat anti‐Mouse IgG (H+L) Alexa Fluor 488 (Invitrogen, A‐11001; 1:500), Donkey anti‐Rabbit IgG (H+L) Alexa Fluor 594 (Invitrogen, A‐32754; 1:500), and Donkey anti‐Goat IgG (H+L) Alexa Fluor 488 (Invitrogen, A‐11055; 1:500). Nuclei were counterstained with SYTOX Deep Red (Invitrogen, S11380; 1:2000) for 30 min at room temperature, followed by PBS washes. Slides were mounted with Mowiol 4‐88 (Sigma‐Aldrich) and stored protected from light. Bright‐field images of histological and IHC‐stained sections were acquired using an Olympus IX83 inverted microscope equipped with a DP73 camera. Confocal images were acquired on a Zeiss LSM 780 microscope using a 25× water‐immersion objective.

For the in vitro assembloid, SafO/FG images were analyzed in Fiji/ImageJ by placing a wide line ROI (200 pixels) approximately perpendicular to the chondral–osteo junction and exporting the distance–intensity profile (Analyze → Plot Profile). For each transect, SafO intensity was normalized to a 0–1 scale using robust min–max scaling (P5–P95). A SafO‐derived transition thickness was defined as the distance between the 80% and 20% points (x20−x80) along the main descending segment of the normalized profile, determined by linear interpolation. For the compartment‐based semiquantification, three regions (chondral layer, interzone, and hPDC layer) were delineated on SafO/FG and Sirius Red images at comparable anatomical levels. Mean staining intensity values were extracted per region using identical settings across samples, and reported as region‐averaged readouts.

### 5.13 SHG Ridge‐Based Fiber Quantification

SHG images acquired for organoid characterization were processed in Fiji (ImageJ, NIH). To reduce high‐frequency noise prior to ridge extraction, images were subjected to Gaussian blur (*σ* = 1) followed by the Despeckle function (median filter) using identical settings for all samples. Quantification was performed on the original intensity images without per‐image contrast rescaling. SHG area fraction (%) was quantified by applying a single global intensity threshold to the SHG channel and calculating the percentage of threshold‐positive pixels within each organoid ROI. The threshold was defined a priori based on background signal measured in SHG‐negative regions and then kept constant for all samples. In addition, ridge‐like structures were extracted from the SHG channel using the Ridge Detection plugin with fixed parameters across all conditions (line width = 3.0, high contrast = 250, low contrast = 80, *σ* = 1.20, lower threshold = 2.00, upper threshold = 5.00, minimum line length = 10, overlap resolution = none). Quantitative metrics were computed per organoid ROI from the exported ridge results, including segment number and total ridge length. Tortuosity was calculated as path length divided by end‐to‐end distance. Segment orientation was treated as axial (0–π), and an alignment index (0–1) was calculated as a length‐weighted orientation coherence. A curvature proxy was computed as cumulative turning along the ridge centerline normalized by ridge length. If no ridges were detected, segment number and total ridge length were recorded as 0, and orientation metrics were reported as not defined (NA).

### 5.14 Depth‐Normalized Four‐Layer Collagen Fiber Orientation Analysis from Polarized Sirius Red Images

Collagen fiber orientation was quantified from Sirius Red–stained sections imaged under polarized light using the Directionality plugin in Fiji/ImageJ (v2.3.0). For each defect, the analysis was performed within the cartilage‐like compartment of the repair tissue. The compartment thickness was defined along a line normal to the articular surface, from the articular surface to the cartilage–subchondral transition, which was identified morphologically as the boundary where the cartilage‐like matrix ends and subchondral trabecular/marrow spaces begin (confirmed on other adjacent histological sections when needed). The compartment thickness was then subdivided into four normalized depth bins (0–25%, 25–50%, 50–75%, and 75–100%). For each depth bin, a rectangular ROI aligned parallel to the local articular surface was selected and analyzed with Directionality (Fourier components; 20 bins; histogram range −90° to 90°; “Build orientation map”, and “Display table” enabled). The resulting orientation histograms were reported as frequency distributions, using identical settings across groups and samples.

### 5.15 Statistical Analysis

Statistical analyses were performed using GraphPad Prism (version 10; GraphPad Software, San Diego, CA, USA), unless otherwise stated. Quantitative continuous data presented as mean ± standard deviation (SD), unless indicated otherwise in the corresponding figure legend. For comparisons between two groups, a two‐tailed unpaired Student's *t*‐test was used. For datasets involving three or more groups, one‐way analysis of variance (ANOVA) was applied as appropriate, followed by Tukey's multiple comparisons test. For paired or repeated measurements obtained from the same biological unit, repeated‐measures one‐way ANOVA followed by Tukey's multiple comparisons test was used as appropriate. Ordinal scoring datasets, including macroscopic and histological scores, were analyzed using the Kruskal–Wallis test followed by Dunn's multiple comparisons test. Bulk RNA‐seq data were processed and analyzed using custom R scripts. Unless otherwise specified, the biological unit used for statistical testing is defined in the corresponding figure legend. When multiple technical subsamples (e.g., serial sections, ROIs, or repeated measurements) were obtained from the same biological unit, values were averaged per biological unit prior to group‐wise statistical analysis to avoid pseudoreplication. No data points were excluded, and no statistical outlier removal was applied. A predefined significance threshold of *p* = 0.05 was used throughout the study. Statistical significance is denoted as ns, not significant; *p < 0.05; **p < 0.01; ***p < 0.001; and ****p < 0.0001. Blinding was not implemented; however, scoring and ROI placement followed predefined anatomical criteria and were complemented by automated quantification using fixed parameters across all groups.

## Conflicts of Interest

The authors declare no conflicts of interest.

## Supporting information




**Supporting File 1**: adma73320‐sup‐0001‐SuppMat.docx.

## Data Availability

The raw and processed RNA sequencing data are deposited at the Gene Expression Omnibus (GEO) under accession code GSE302312. The numerical source data underlying all main and supporting figures (Excel, “Updated Source Data”), the cross‐reference index, and the original, unprocessed images (“Updated Raw Images” ZIP archives) are deposited in Zenodo (https://doi.org/10.5281/zenodo.20070283). Any other data supporting the findings of this study are available from the corresponding author upon reasonable request.
