# Supplementary material for: Synchronizing the Osteochondral Regeneration Process through Spatial Patterning of Stable and Hypertrophic Cartilage Organoids
Source: Adv Mater. 2026 May 8;38(33):e16189. doi: 10.1002/adma.202516189 (PMC13261385; doi:10.1002/adma.202516189)
Supplement: Supplementary file 1 — Supporting File 1: adma73320‐sup‐0001‐SuppMat.docx. [file ADMA-38-e16189-s001.docx]

Supporting Information

**Synchronizing the Osteochondral Regeneration Process through Spatial Patterning of Stable and Hypertrophic Cartilage Organoids**

Liuqi Peng^1,2^, Isaak Decoene^1,2^, Hanna Svitina^1,2^, Ioannis Papantoniou^1,2,^*

^1^ Prometheus the Translational Division of Skeletal Tissue Engineering, Leuven R&D, KU Leuven, O&N1, Herestraat 49, PB 813, 3000 Leuven, Belgium

^2^ Skeletal Biology and Engineering Research Center, Department of Development and Regeneration, KU Leuven, O&N1, Herestraat 49, PB 813, 3000 Leuven, Belgium

*Corresponding author (E-mail: ioannis.papantoniou@kuleuven.be)

# **Supplementary Figures**


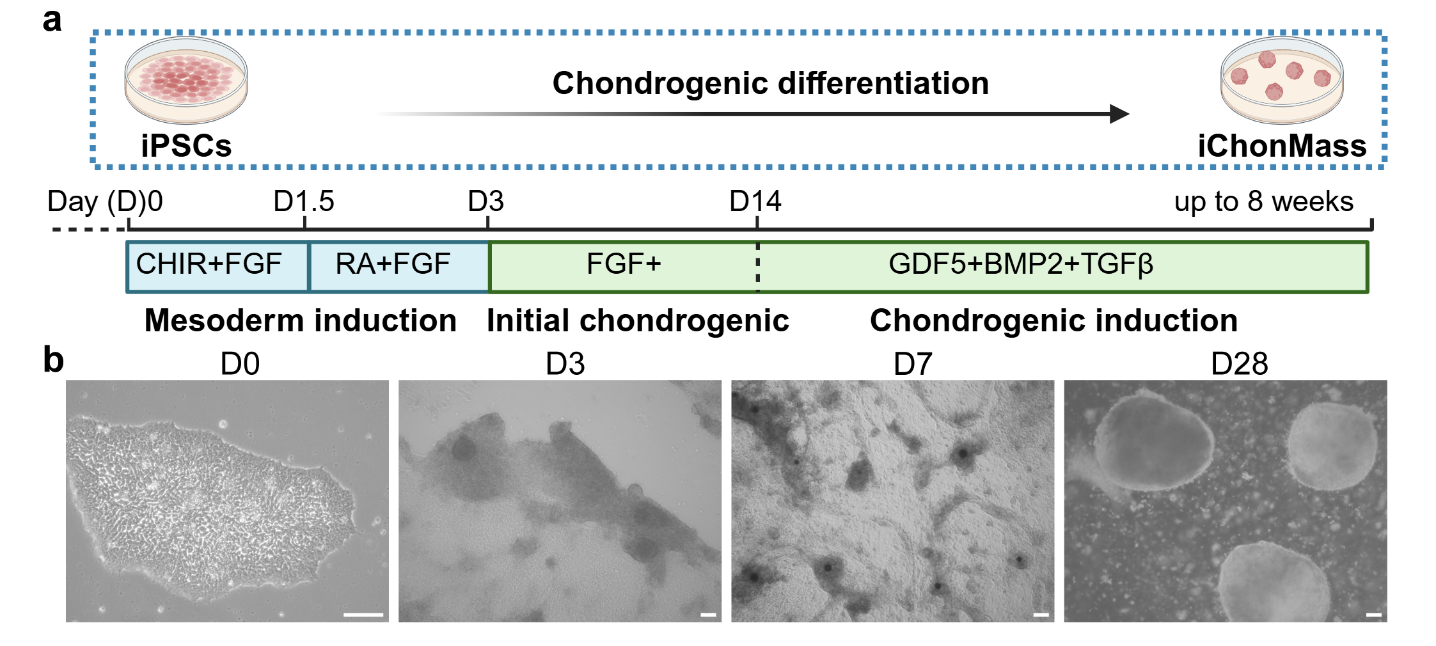


**Supplementary Figure 1**. (a) Schematic of the human induced pluripotent stem cell (iPSC; BIONi010-C) chondrogenic differentiation protocol. (b) Morphology of iPSC cultures at different stages of differentiation (scale bar: 200 µm).


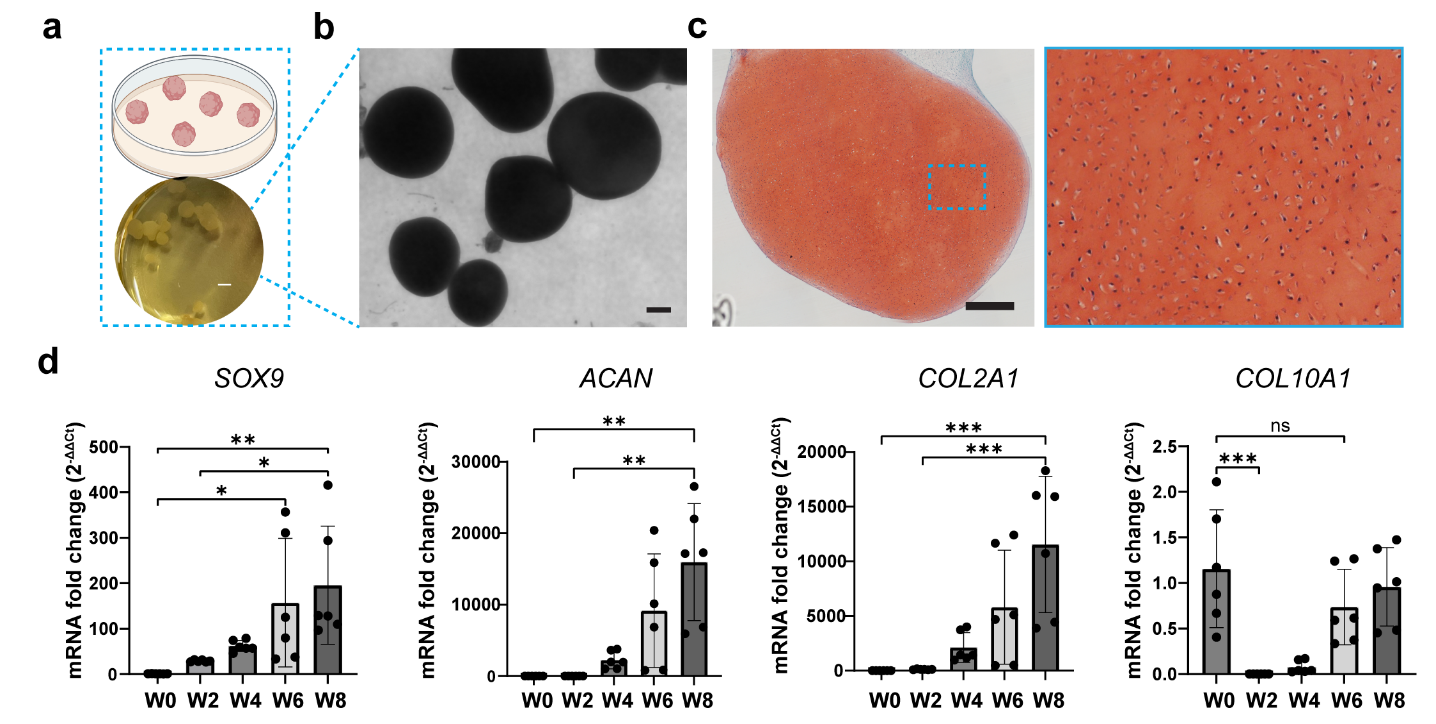


**Supplementary Figure 2**. (a) Schematic of iChonMass (top) and iChonMass cultured in a 6-well plate (bottom). (b) Bright-field image of iChonMass at week 8. (c) Safranin O/Fast Green (SafO/FG) staining of iChonMass at week 8 with a higher-magnification view shown on the right. Scale bars: 2 mm in (a) and 500 µm in (b–c). (d) Quantification of gene expression changes during iPSC chondrogenic differentiation, normalized to week 0 (W0). Data presented as mean ± SD; n = 6 biological replicates; group differences were assessed using one-way ANOVA followed by Tukey’s multiple comparisons test. ns, not significant; **p* < 0.05; ***p* < 0.01; ****p* < 0.001; *****p* < 0.0001.


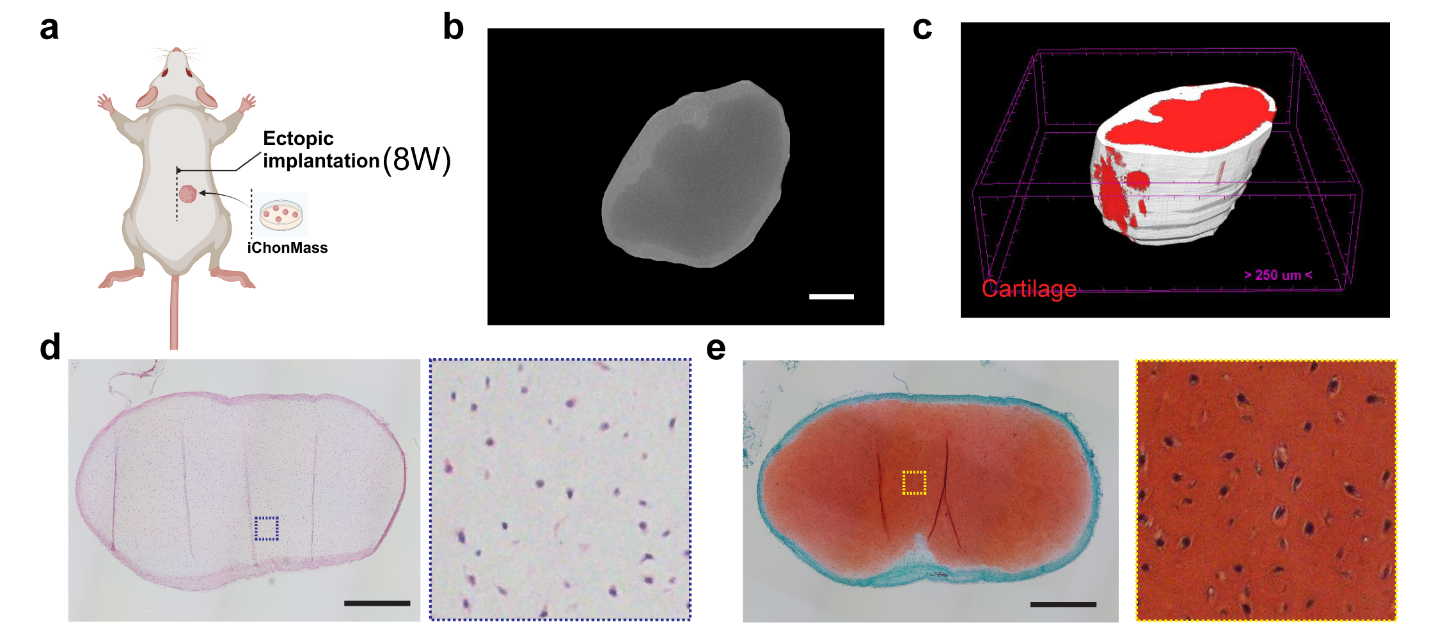


**Supplementary Figure 3**. (a) Schematic of 8-week subcutaneous implantation of iChonMass. (b) Representative nanoCT image of the explant with Hexabrix contrast at 8 weeks (dark grey: cartilage; light grey: other tissue). (c) 3D rendering of the explant (red: cartilage; white: surrounding connective tissue). (d) Hematoxylin and eosin (H&E) and (e) Safranin O/Fast Green (SafO/FG) staining of the explants, showing maintenance of the chondrogenic phenotype. Scale bar: 500 µm.


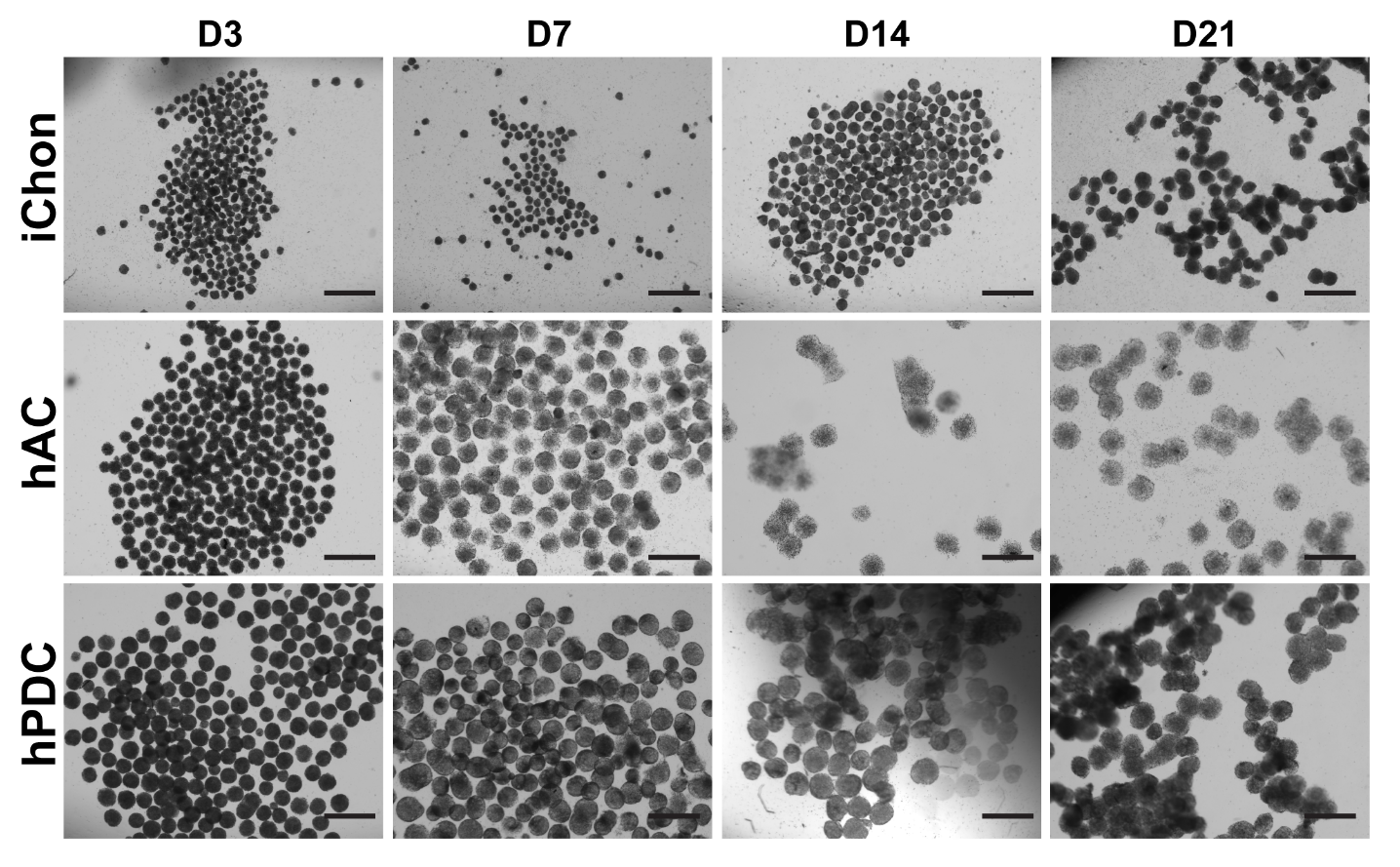


**Supplementary Figure 4**. Representative bright-field images of different organoid types (iChon, hAC, and hPDC) collected in the well plate. Scale bar: 1 mm.


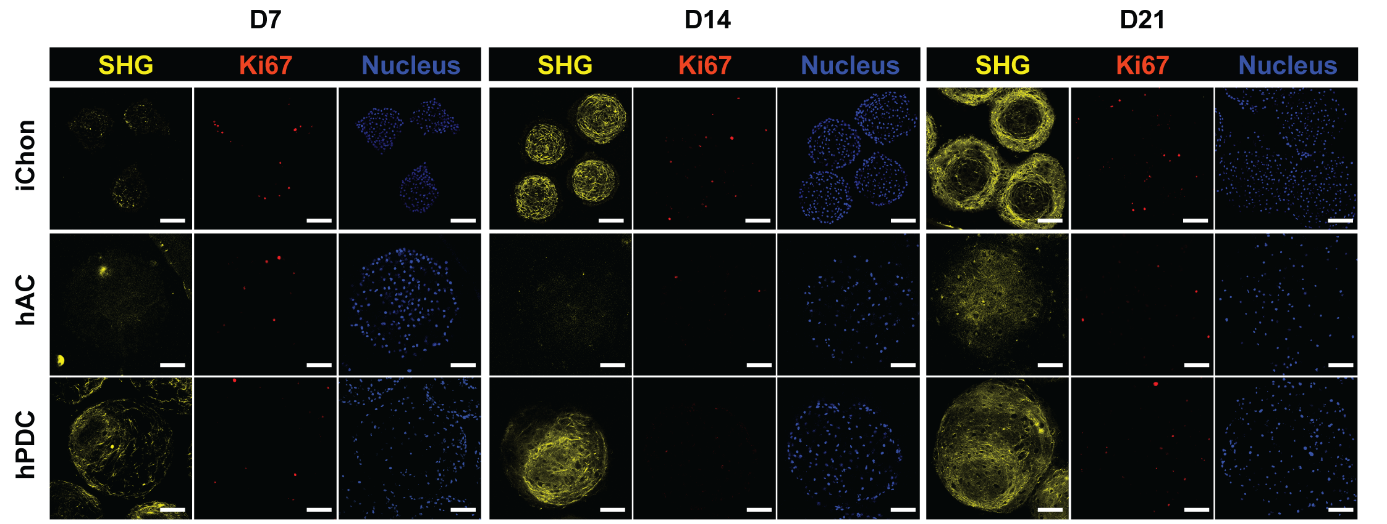


**Supplementary Figure 5**. Representative single-channel images of Ki67 (red), SHG collagen signal (yellow), and nuclei (blue) in iChon, hAC, and hPDC organoids at day 7, 14, and 21. Scale bar: 100 µm.


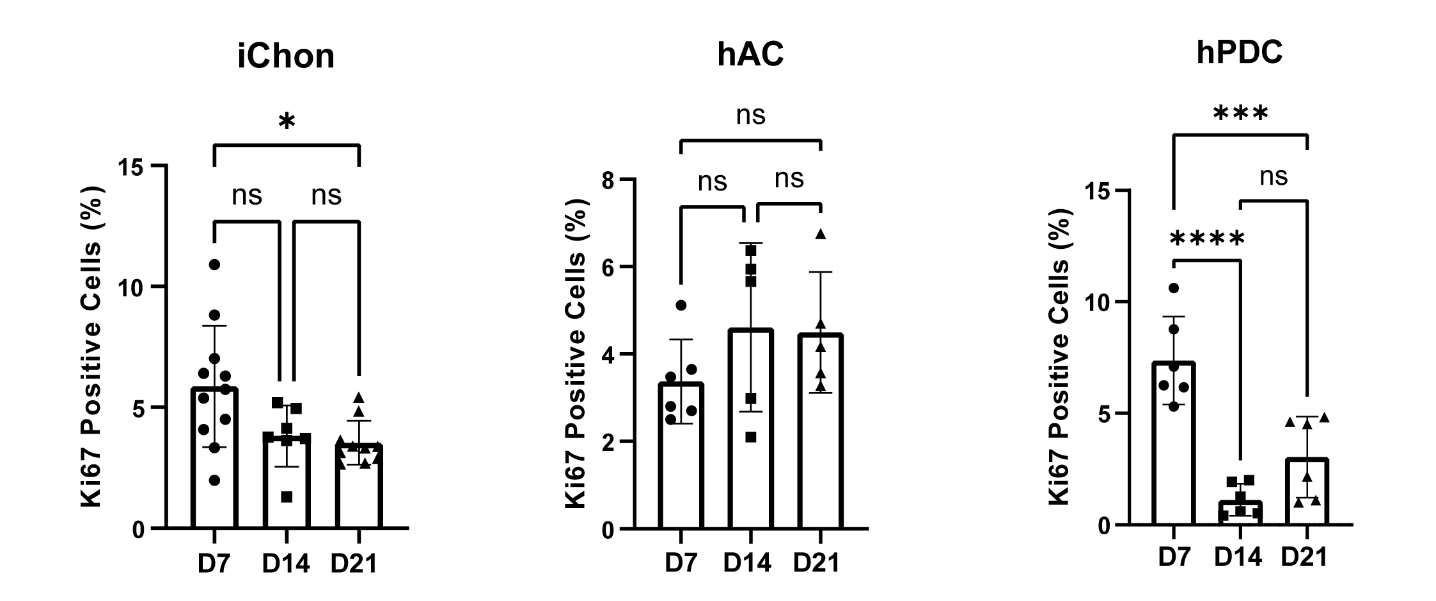


**Supplementary Figure 6**. Semi-quantification of Ki67 positive cells in iChon, hAC, and hPDC organoids at day 7, 14, and 21. Data presented as mean ± SD; n > 5, each dot represents one organoid; Within each group, time-course differences were assessed using one-way ANOVA followed by Tukey’s multiple comparisons test. ns, not significant; **p* < 0.05; ***p* < 0.01; ****p* < 0.001; *****p* < 0.0001.


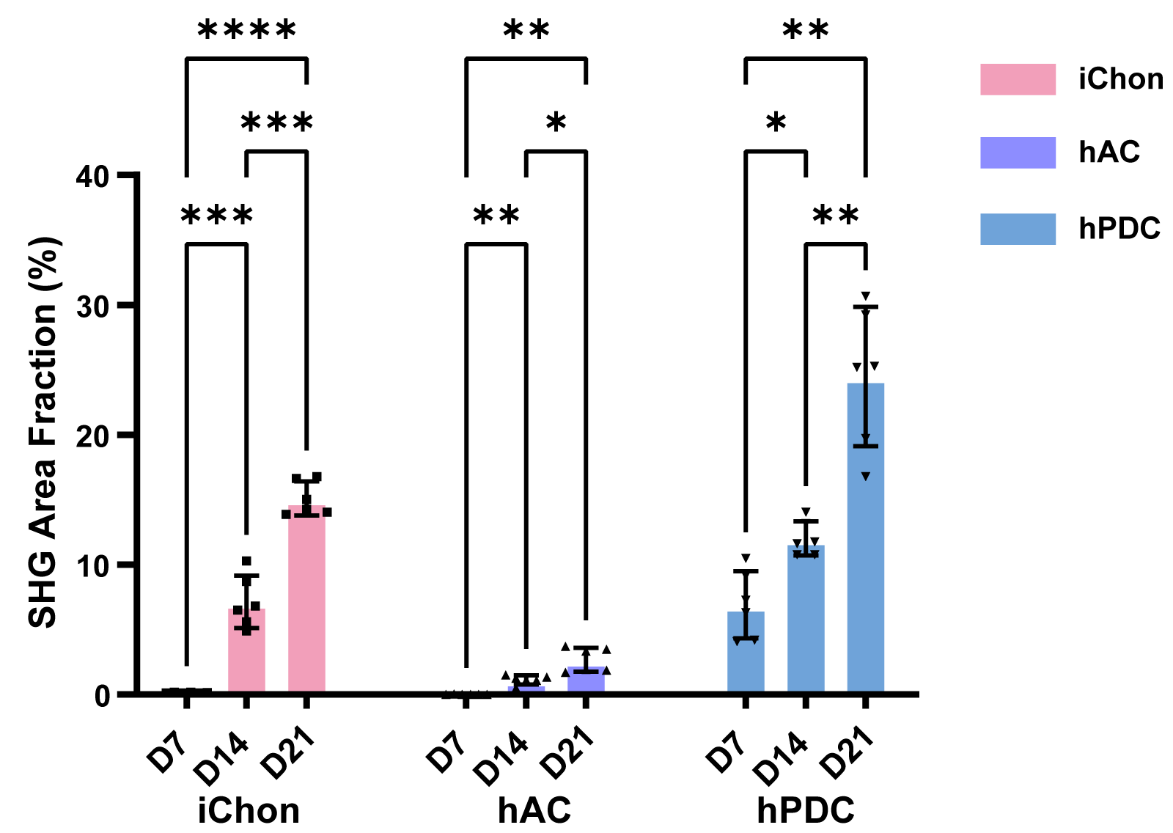


**Supplementary Figure 7**. Quantification of SHG area fraction (%) in iChon, hAC, and hPDC microtissues at Day 7, 14, and 21. Data presented as mean ± SD, n = 6 organoids per group per time point, *p* values are calculated using one-way ANOVA followed by Tukey’s multiple comparison test. ns: not significant, **p* < 0.05, ***p* < 0.01, ****p* < 0.001, *****p* < 0.0001.


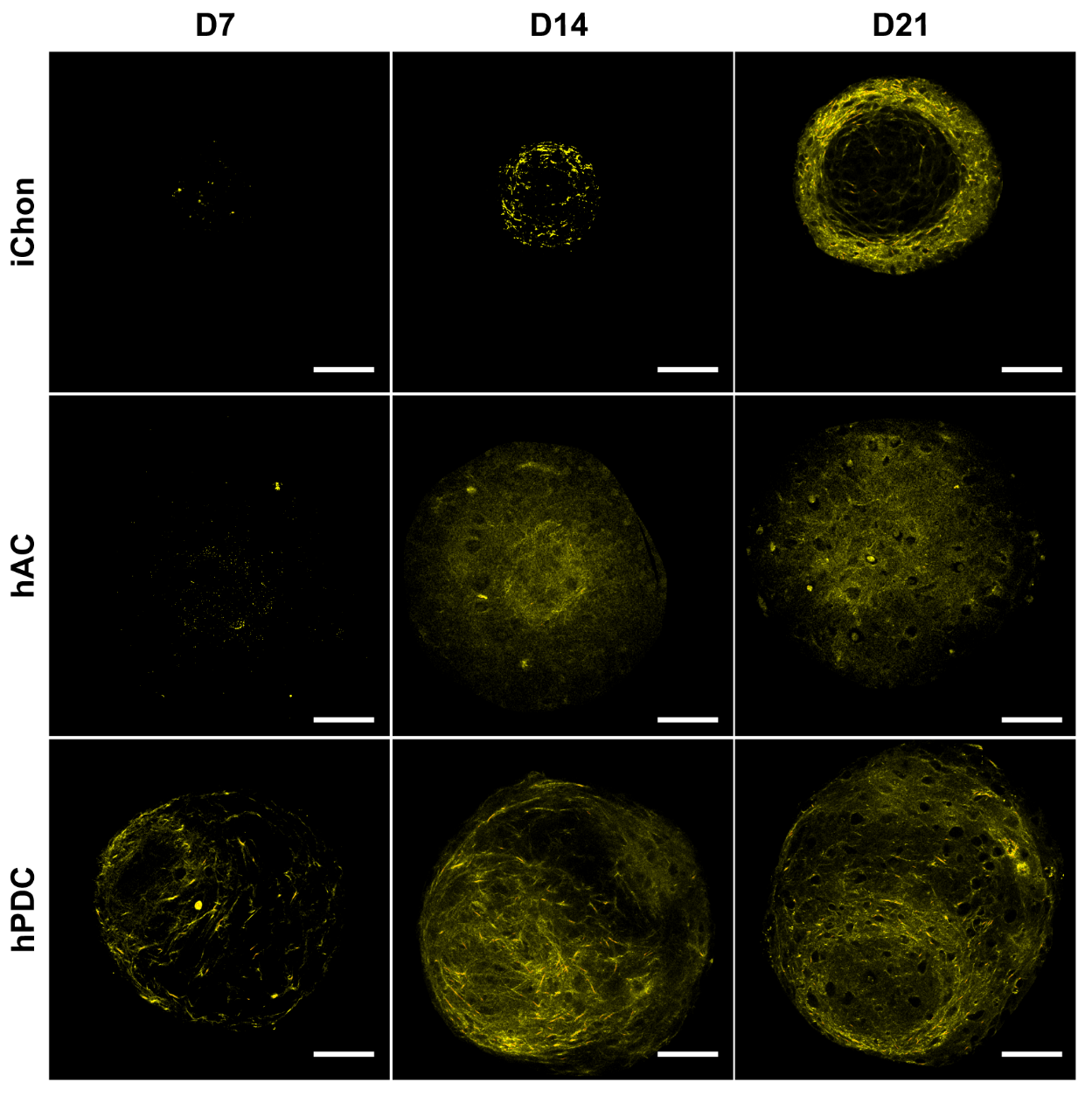


**Supplementary Figure 8**. Representative ridge-detection overlays generated from the SHG channel using fixed Ridge Detection parameters to illustrate ridge centerlines (red line) used for fiber-like feature quantification (scale bars: 100 µm).


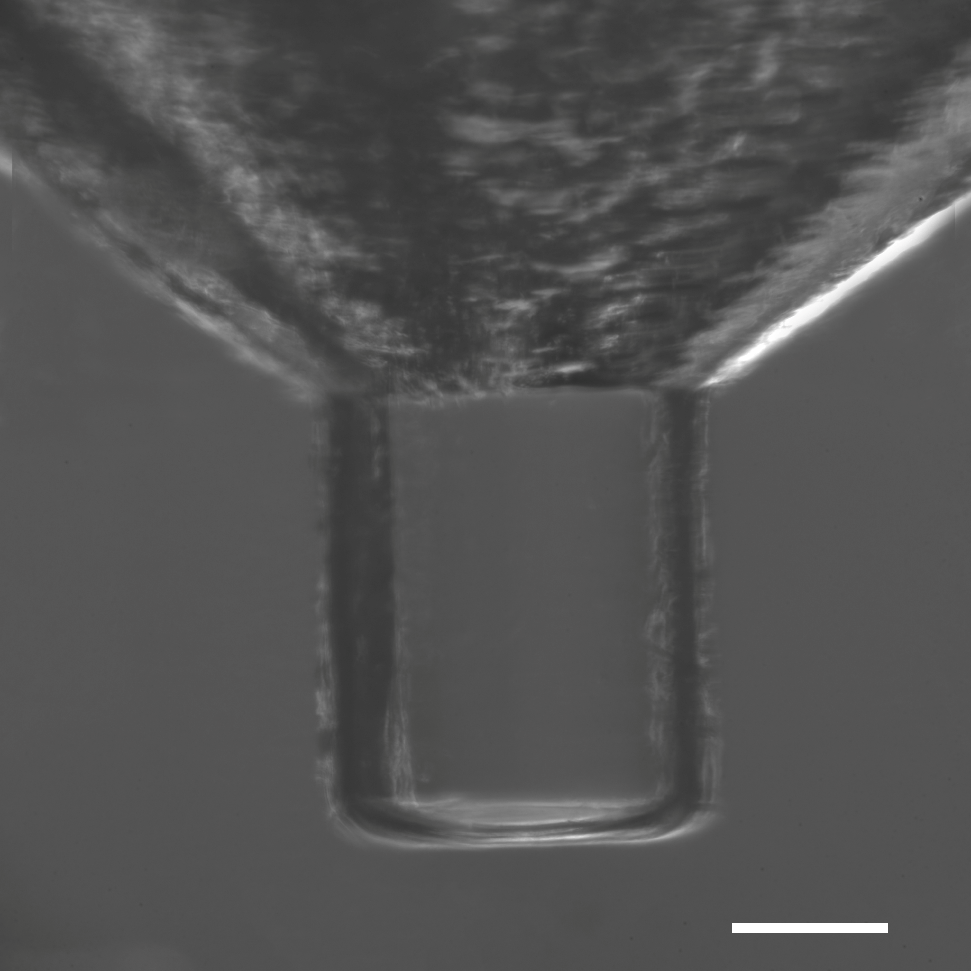


**Supplementary Figure 9.** Representative bright-field image of the customized agarose mold with diameter 2 mm for osteochondral-like assembloid formation (scale bar: 1 mm).


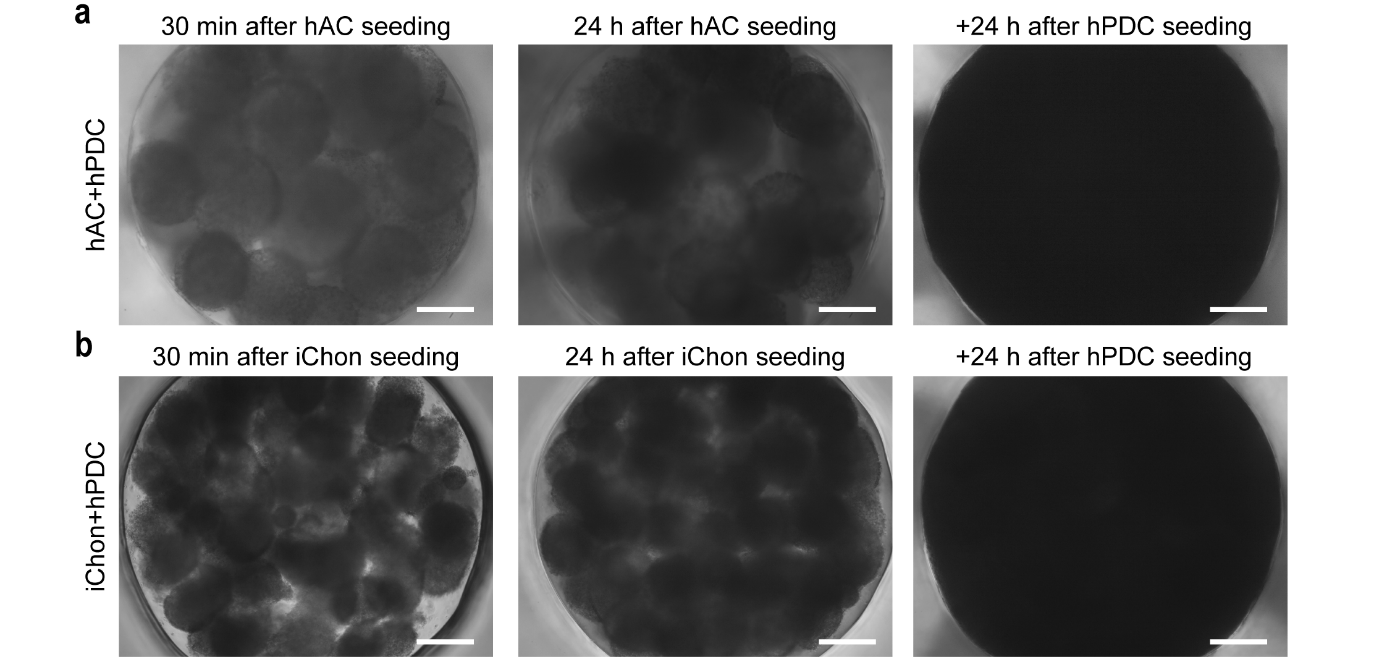


**Supplementary Figure 10.** Representative bright-field images of sequential fusion during assembloid assembly (top view). The snapshots were acquired at 30 min and 24 h after seeding the chondral-layer organoids (hAC or iChon), and 24 h after subsequent hPDC seeding (48 h total). (scale bar: 500 µm).


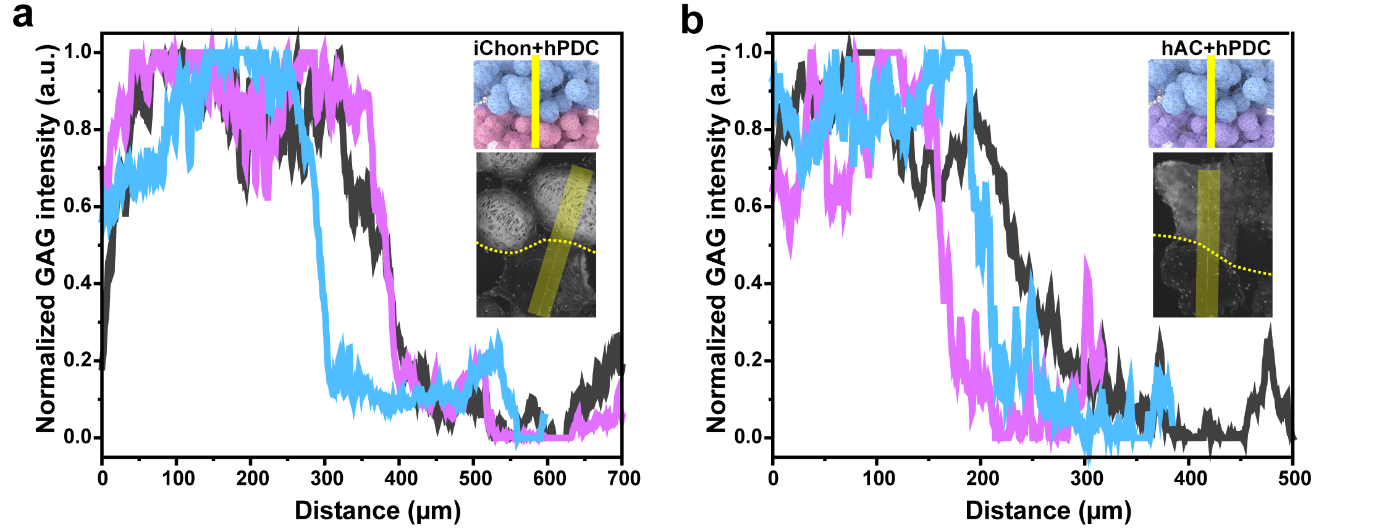


**Supplementary Figure 11.** Normalized SafO line-scan profiles across the assembloid junction. (a) iChon+hPDC and (b) hAC+hPDC. Three representative line transects were sampled across the chondral–osteo junction and plotted as normalized SafO (GAG) intensity versus distance. Insets show the schematic and an example transect placement.

**
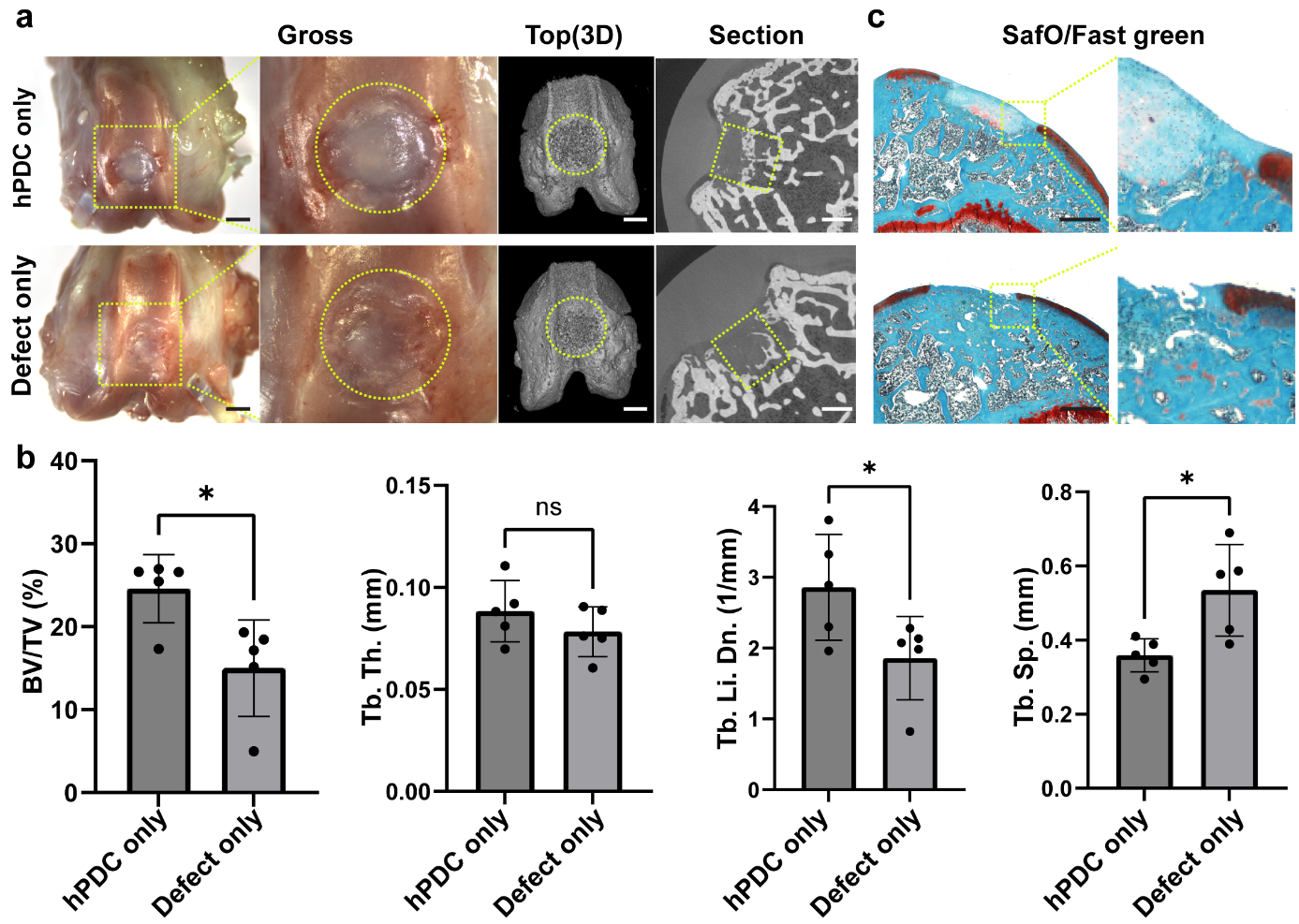
**

**Supplementary Figure 12.** *In vivo* evaluation of hPDC-only assembloid repair at 8 weeks post-implantation. (a) Gross images and top-view 3D reconstructions, and representative sectional nanoCT views of hPDC-only and defect-only groups at 8 weeks post-implantation (scale bar: 500 µm). (b) Quantitative nanoCT analysis of bone volume fraction (BV/TV), trabecular thickness (Tb.Th), trabecular linear density (Tb.Li.Dn), and trabecular separation (Tb.Sp) within the defect VOI. Data presented as mean ± SD, n = 5 animals per group, group differences were assessed using an unpaired two-tailed t-test. ns, not significant; **p* < 0.05. (c) Safranin O/Fast Green staining of the explants and the zoom-in images for both groups (scale bar: 500 µm).


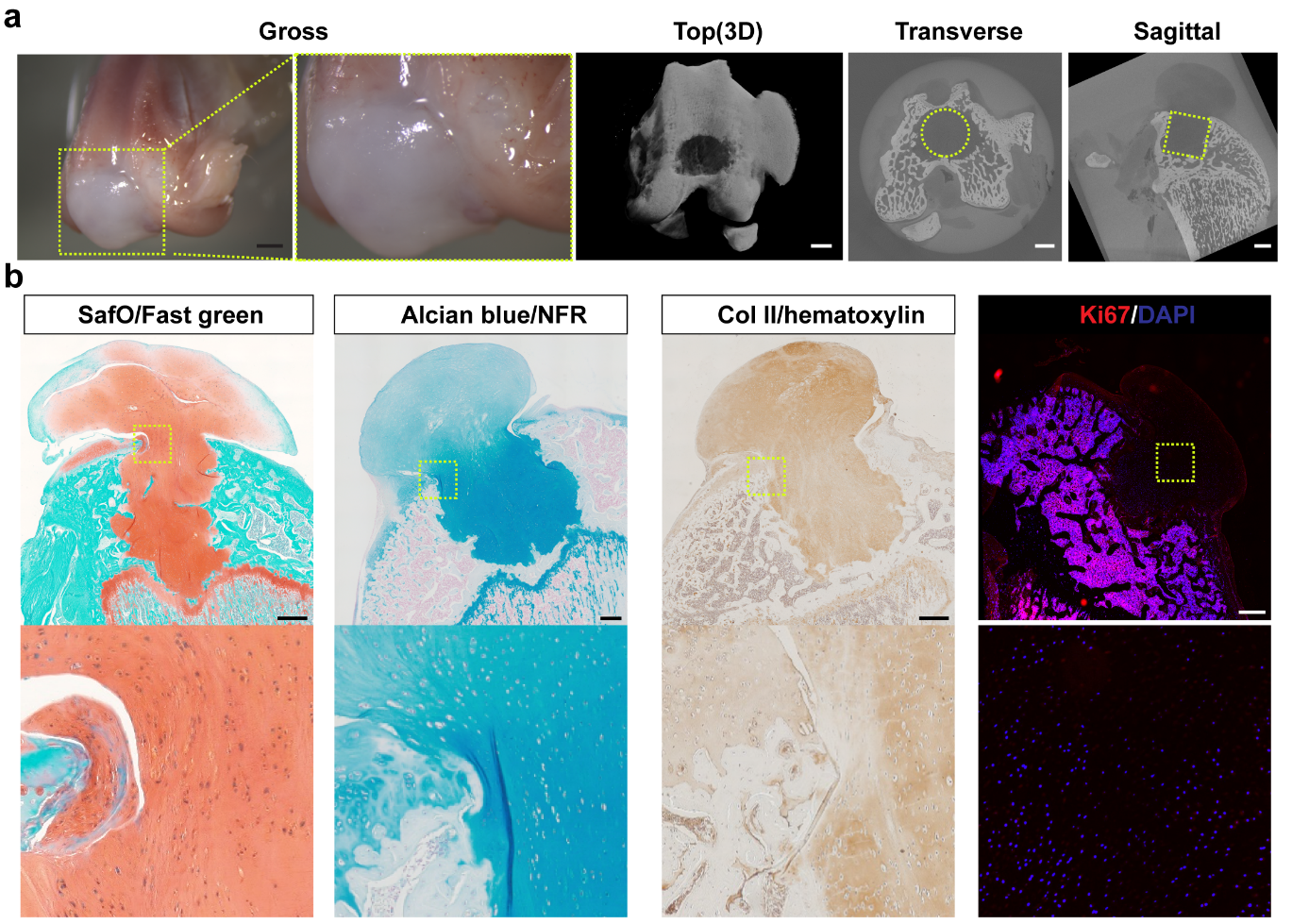


**Supplementary Figure 13.** *In vivo* evaluation of iChon-only repair at 16 weeks post-implantation. (a) Gross images and top-view 3D reconstructions of iChon-only assembloid at 16 weeks post-implantation, together with transverse and sagittal views of the iChon-only group. (b) Histological staining of explants, including Safranin O/Fast Green, Alcian Blue, collagen type II (COL II) immunohistochemistry, and Ki67/DAPI immunofluorescence, to assess cartilage matrix deposition and proliferative activity. All panels scale bar: 500 µm.


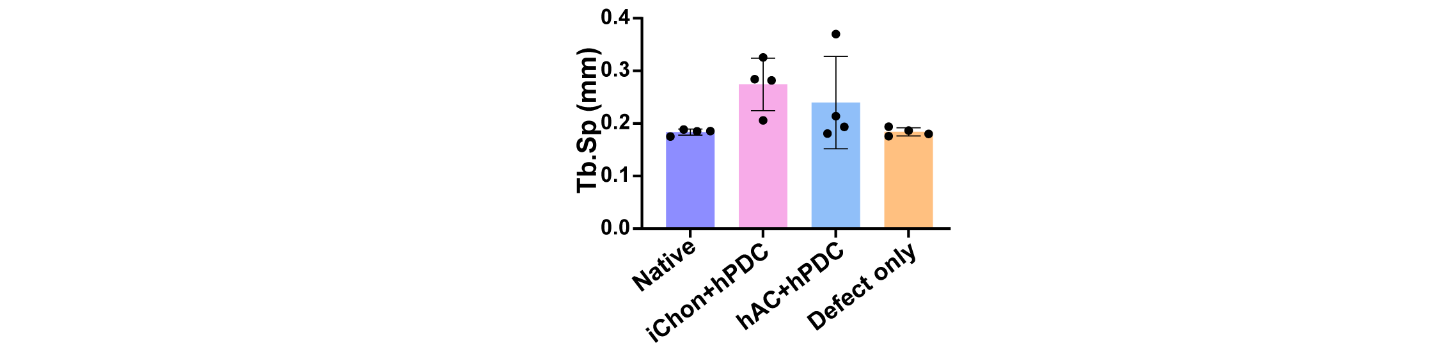


**Supplementary Figure 14.** Quantitative nanoCT analysis of trabecular separation (Tb.Sp) in regenerated subchondral bone for all groups. Data presented as mean ± SD; n = 4 animals per group, each dot represents one animal; group differences were assessed using one-way ANOVA followed by Tukey’s multiple comparisons test. ns, not significant; **p* < 0.05; ***p* < 0.01; ****p* < 0.001; *****p* < 0.0001.


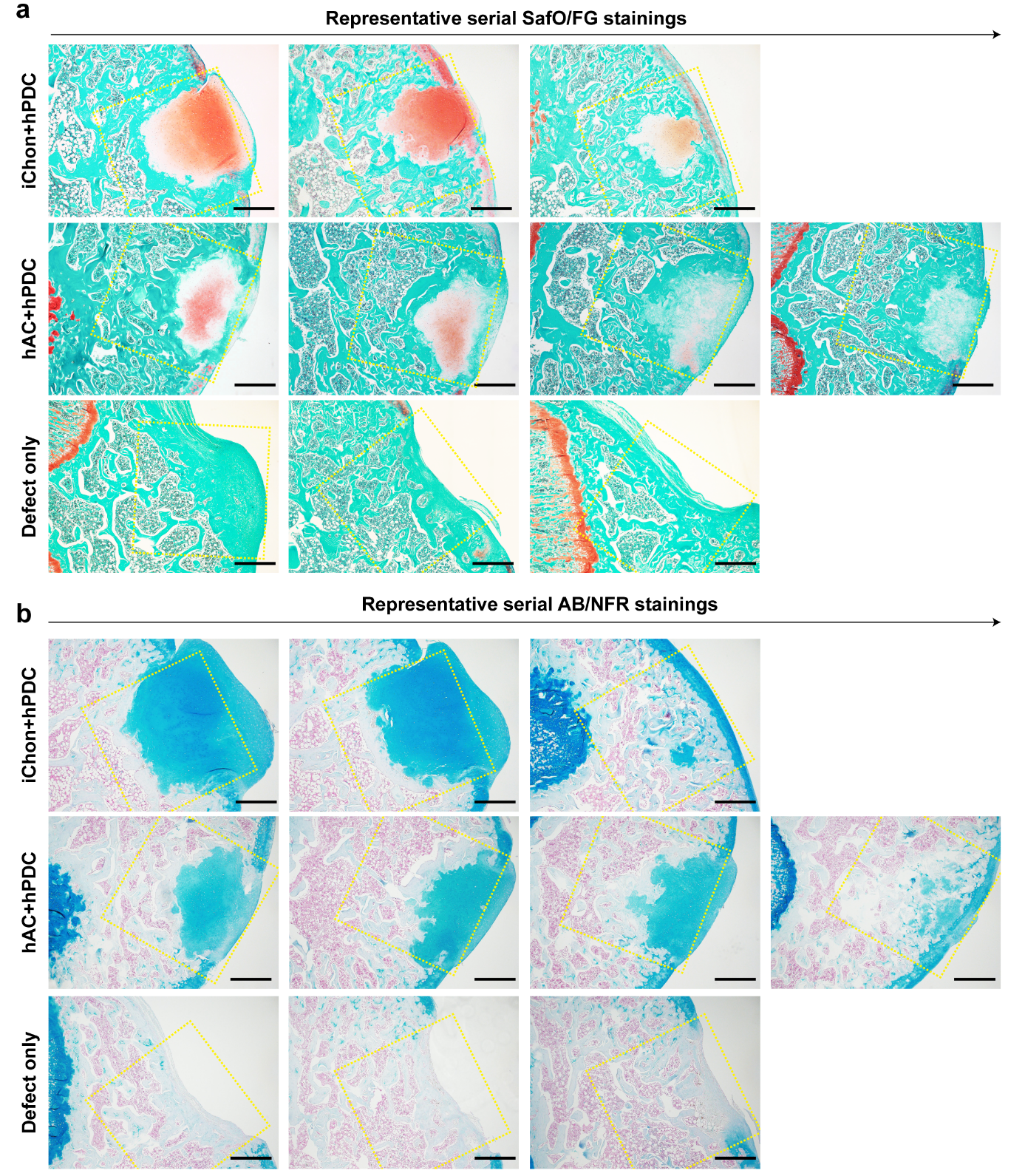


**Supplementary Figure 15**. Representative serial sections of explants from the iChon+hPDC, hAC+hPDC, and defect-only groups stained with (a) Safranin O/Fast Green and (b) Alcian Blue/Nuclear Fast Red. Images are shown in serial order (left to right), with the displayed planes separated by approximately 100 µm. The yellow dashed line indicates the defect area. Scale bar: 500 µm.


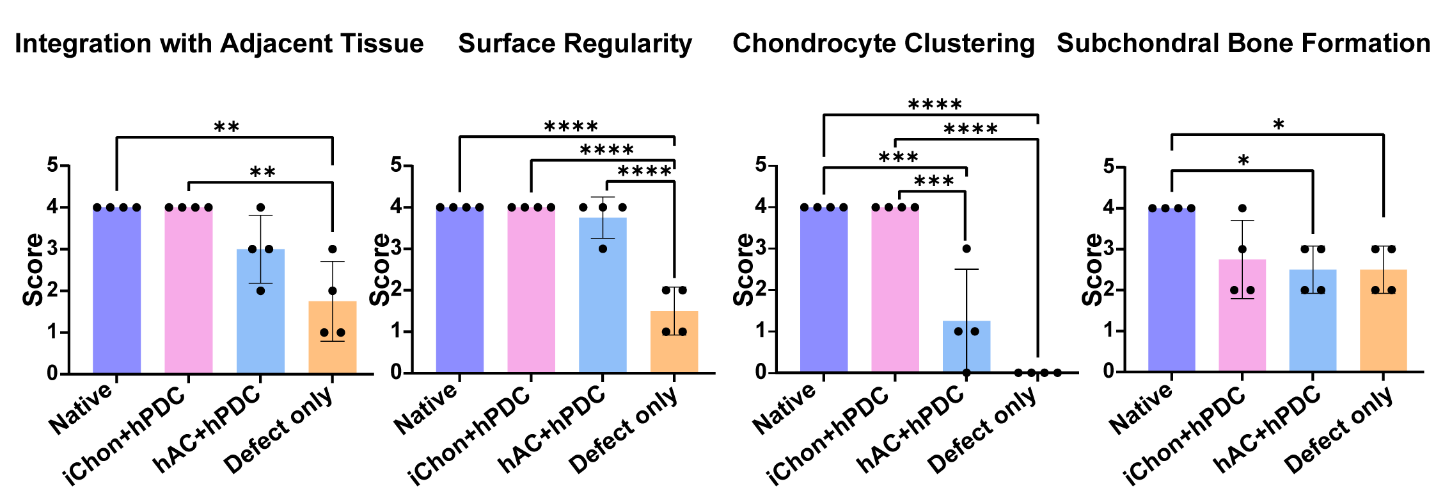


**Supplementary Figure 16.** Subcategory analysis score of integration with adjacent tissue, surface regularity, chondrocyte clustering and subchondral bone formation based on modified O’Driscoll histological assessment for osteochondral repair quality. Data presented as mean ± SD; n = 4 animals per group, each dot represents one animal; group differences were assessed using the Kruskal–Wallis test followed by Dunn’s multiple comparisons test. ns, not significant; **p* < 0.05; ***p* < 0.01; ****p* < 0.001; *****p* < 0.0001.


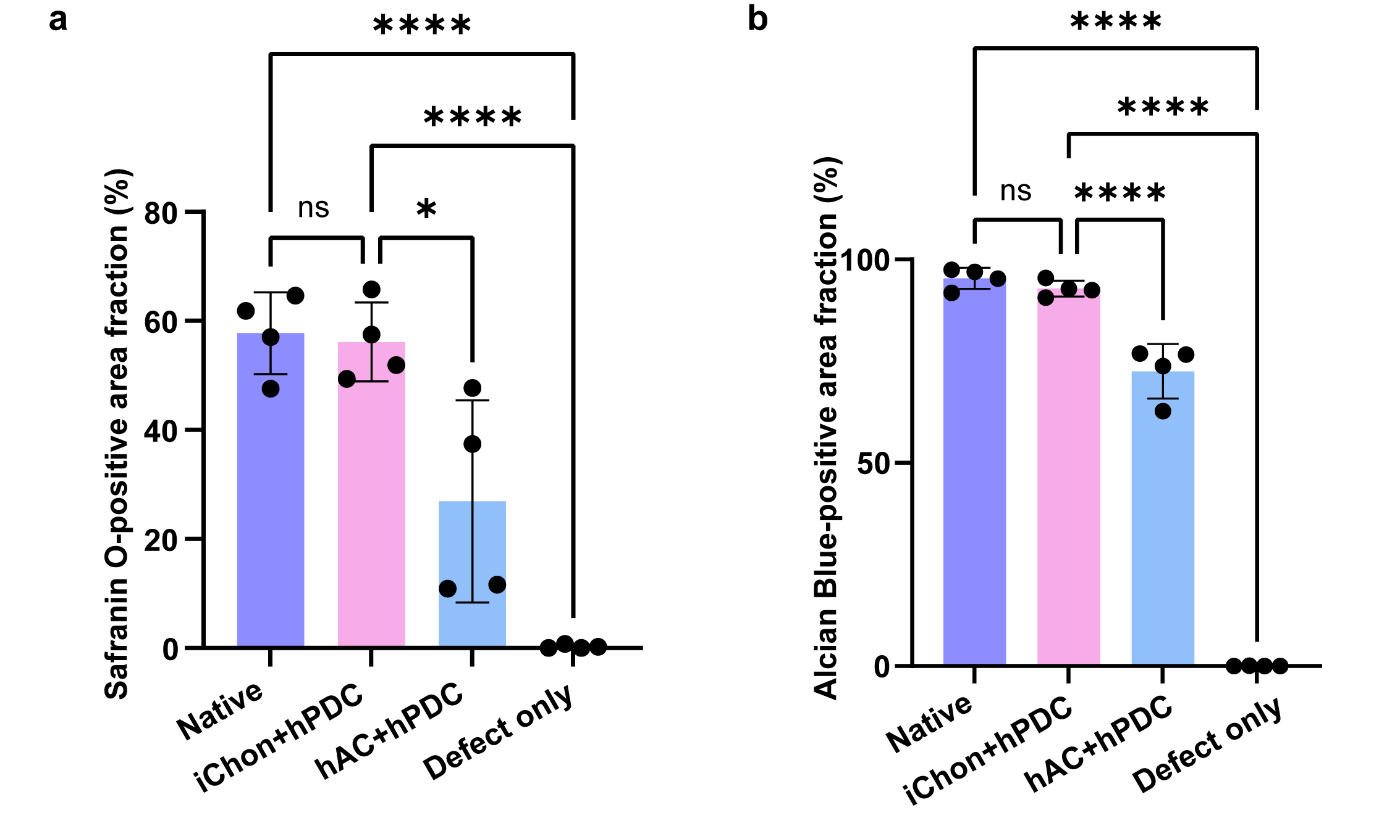


**Supplementary Figure 17**. Semi-quantification of cartilage-associated histological staining within the native-referenced cartilage-like repair compartment. (a) Safranin O-positive area fraction (%) and (b) Alcian Blue-positive area fraction (%) within the native-referenced cartilage-like repair compartment. Within the defect region, the native-referenced cartilage-like repair compartment was defined according to the adjacent native cartilage plane. Data presented as mean ± SD; n = 4 animals per group, each dot represents one animal; Group differences were assessed using one-way ANOVA followed by Tukey’s multiple comparisons test. ns, not significant; **p* < 0.05; ***p* < 0.01; ****p* < 0.001; *****p* < 0.0001.


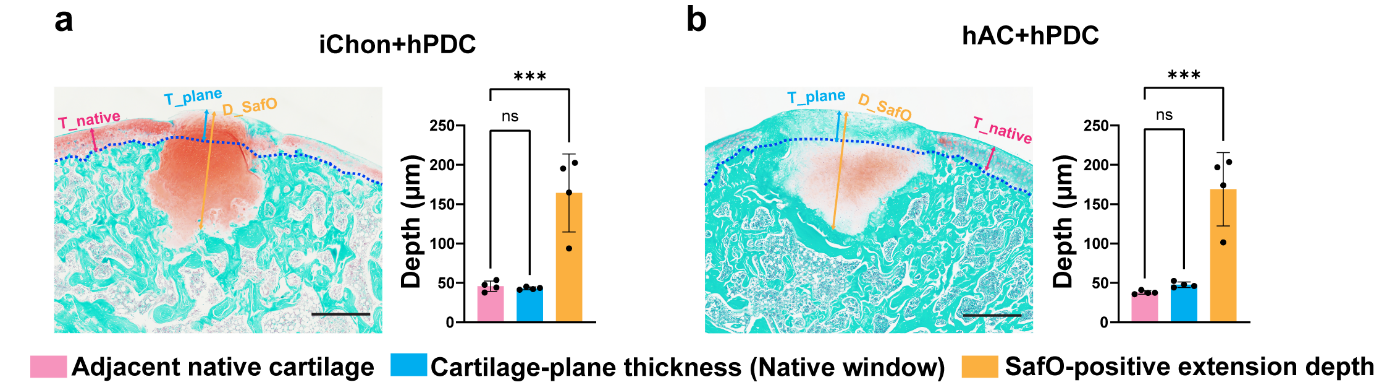


**Supplementary Figure 18.** Native-referenced cartilage-plane thickness and SafO-positive extension depth in vivo. Representative SafO/FG sections (central plane) from (a) iChon+hPDC and (b) hAC+hPDC defects at 16 weeks illustrate measurement of adjacent native cartilage thickness (T_native), cartilage-plane thickness within the defect (T_plane; native window), and SafO-positive extension depth into the deep compartment (D_SafO; measured to the deepest clearly SafO-positive region). Data presented as mean ± SD; n = 4 animals per group, each dot represents one animal, and each value corresponds to the mean of six measurements for the indicated category within that animal; Group differences were assessed using repeated-measures one-way ANOVA followed by Tukey’s multiple comparisons test. ns, not significant; **p* < 0.05; ***p* < 0.01; ****p* < 0.001; *****p* < 0.0001. Scale bar: 500 µm.


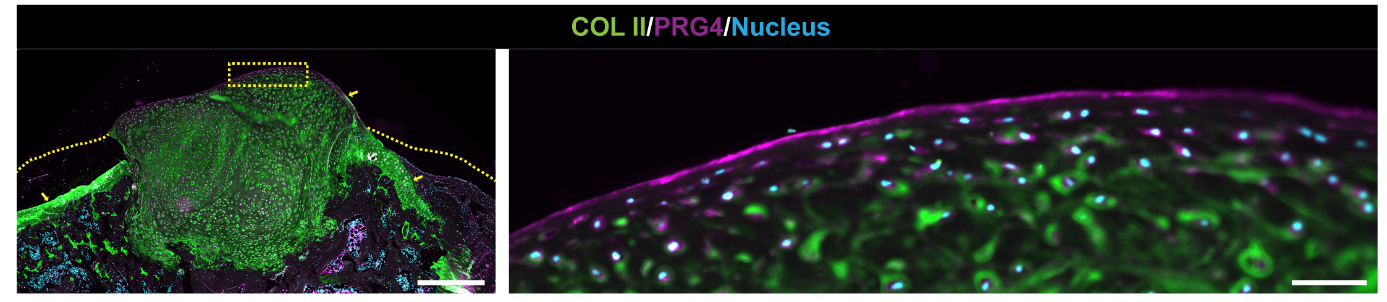
**Supplementary Figure 19**. Representative immunofluorescence staining of PRG4 (Magenta), type II collagen (COL II, green) and nuclei (DAPI, cyan). Scale bars: 500 µm (overview) and 50 µm (zoom-in).


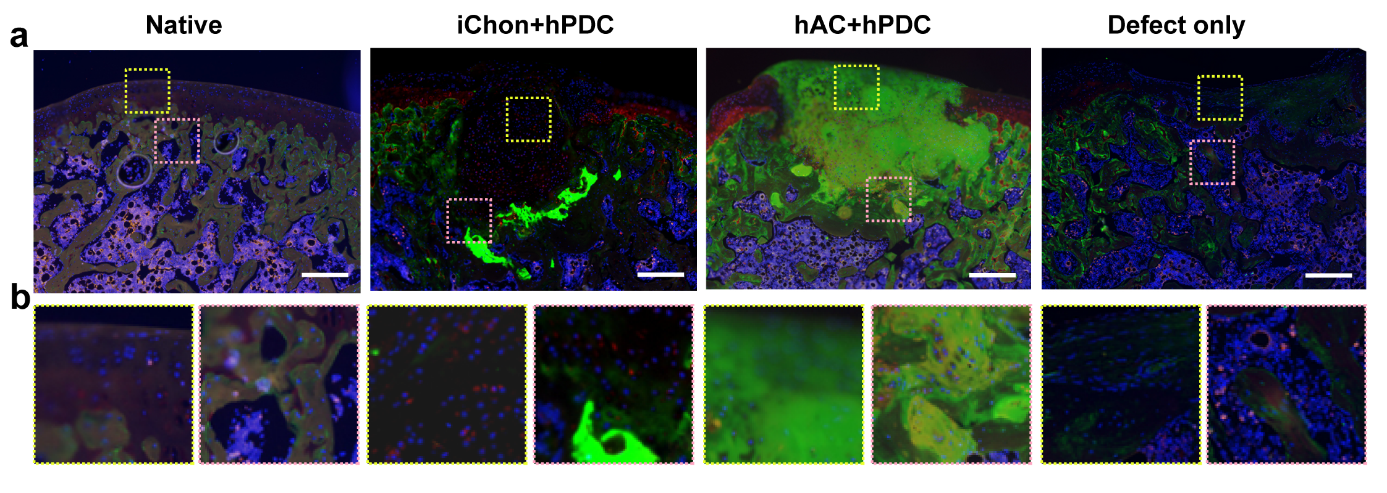


**Supplementary Figure 20**. Representative immunofluorescence staining of type I collagen (COL I, green), type II collagen (COL II, red) and nuclei (DAPI, blue) across different groups (scale bar: 500 µm).


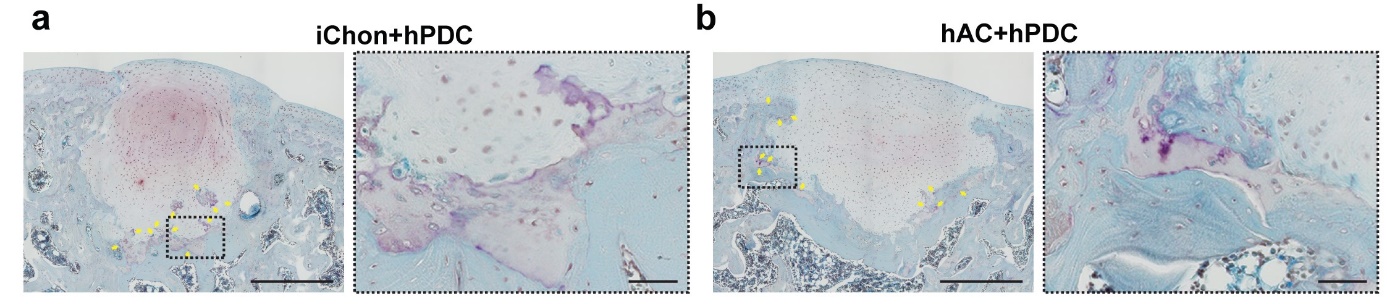


**Supplementary Figure 21**. Tartrate-resistant acid phosphatase (TRAP) staining indicates remodeling-associated resorptive activity at the defect base/subchondral compartment at 16 weeks *in vivo*. Representative TRAP staining of (a) iChon+hPDC and (b) hAC+hPDC groups. Dashed rectangles indicate the regions displayed at higher magnification. Yellow arrowheads mark TRAP-positive areas/cells located along trabecular/subchondral surfaces adjacent to the repaired region. (scale bars: 500 µm, and zoom-in image scale bar: 50 µm).


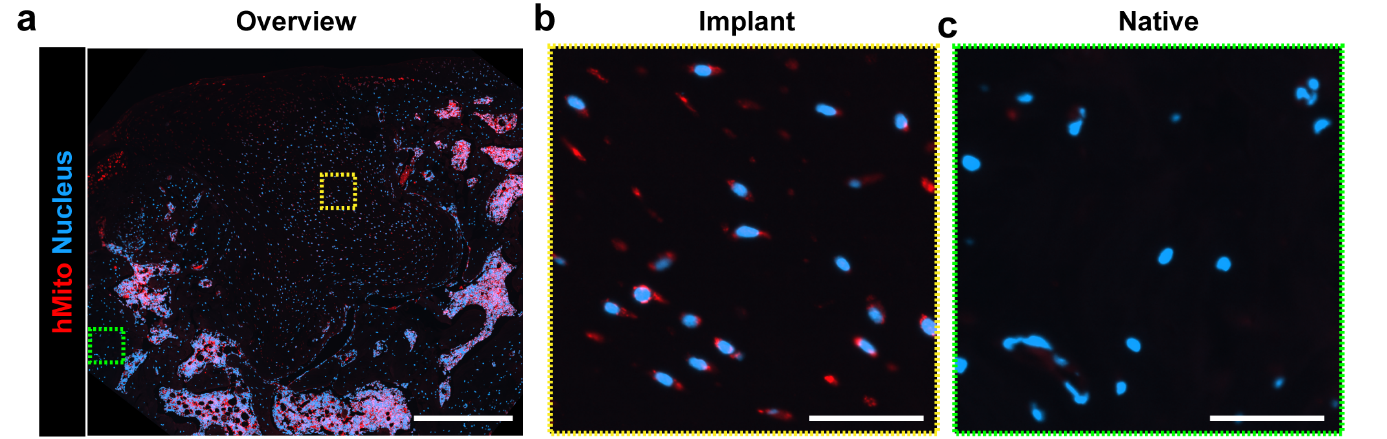


**Supplementary Figure 22**. Human-specific mitochondria staining indicates donor-associated human signal in the repair tissue at 16 weeks. (a) Overview of the repair region stained for human mitochondria (hMito, red) and nuclei (DAPI, cyan) in iChon+hPDC group. Boxes indicate regions magnified in (b) from the implant region showing cell-associated hMito signals, and (c) Adjacent native region from the same section shows no specific hMito signal. Scale bars: 500 µm in (a) and 50 µm in (b–c).


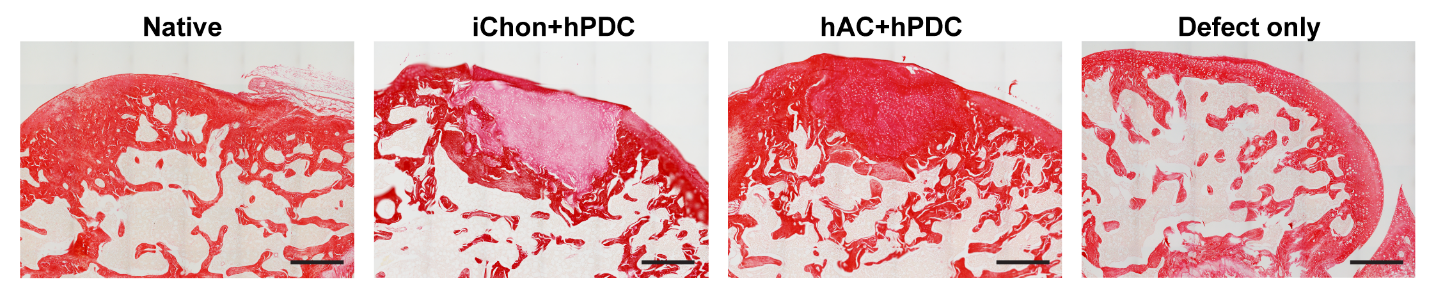


**Supplementary Figure 23.** Representative Sirius Red staining images of native control, iChon+hPDC, hAC+hPDC, and Defect-only groups at 16 weeks post-implantation (Scale bar: 500 µm).


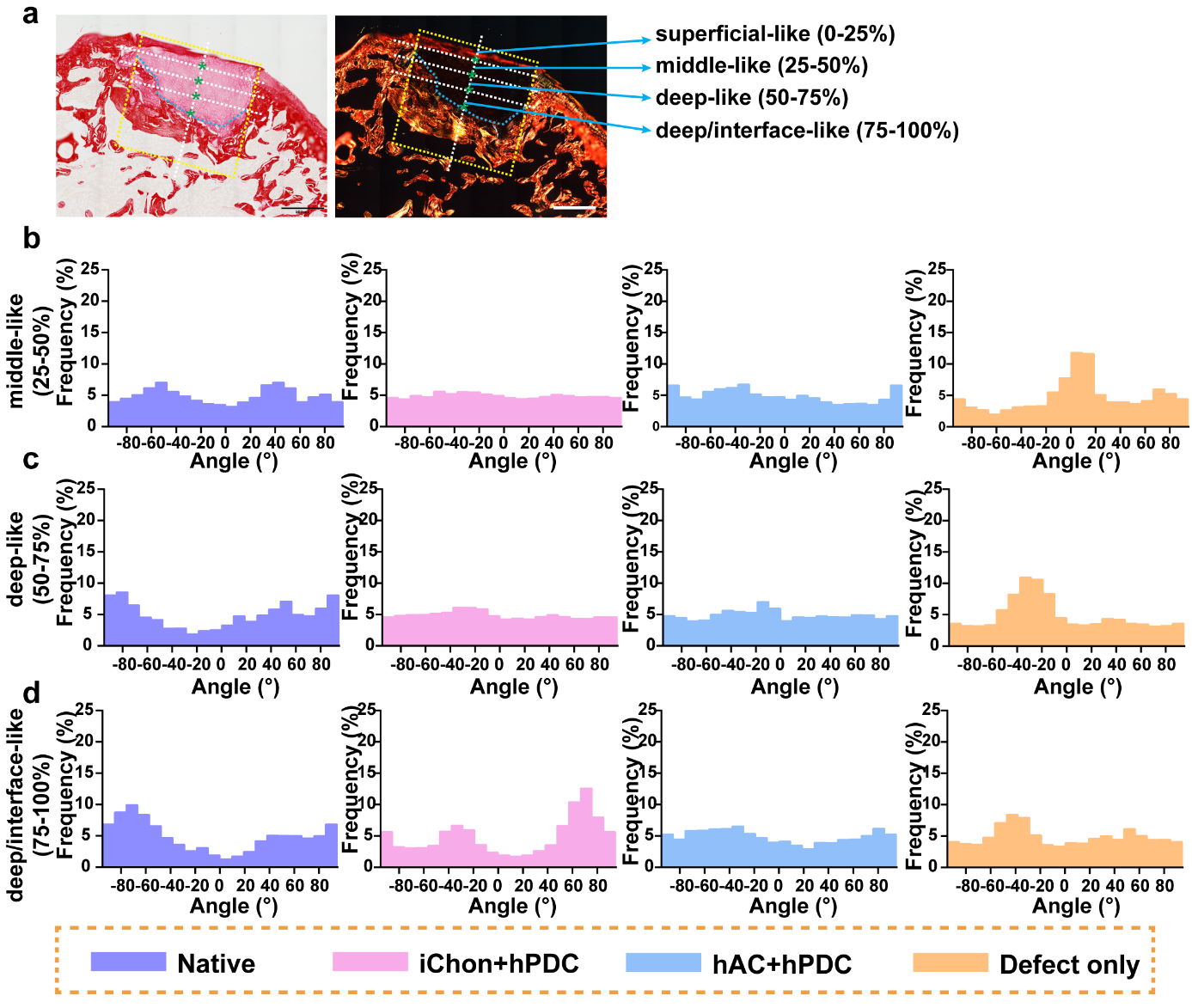


**Supplementary Figure 24**. Depth-wise collagen fiber orientation analysis of the cartilage-like compartment. (a) Representative definition of the analysis region and depth bins. The cartilage-like compartment within the repair region was segmented from the articular surface to the cartilage–subchondral transition, and subdivided into normalized depth bins (0–25%, 25–50%, 50–75%, and 75–100% of the compartment thickness). The superficial bin (0–25%) is shown in the main manuscript (Fig. 6b–c); the remaining bins are provided here to address depth-dependent (four-layer) comparison. (b–d) Directionality histograms of collagen fiber orientation for the 25–50% (b), 50–75% (c), and 75–100% (d) depth bins in native cartilage and in the repair tissue of the indicated groups, quantified from Sirius Red images acquired under polarized light using the same analysis settings as in the main figures. These bins are operational and do not correspond directly to the mature native cartilage layers. n = 4 animals per group; Directionality histograms were computed per animal (averaged across ROIs/serial sections) and then summarized at group level.

# **Supplementary Tables**

**Supplementary Table 1.** Ridge-based quantification of SHG-detected collagen fiber-like features in organoids. Data are shown as mean ± SD with organoid ROI as the statistical unit (n = 6). When no fibers were detected, Total traced length and Segment number were recorded as 0, while shape/orientation metrics are not defined (NA).

| **Group** | **Day** | **n (organoid ROIs)** | **Traced segments (N)** | **Total traced length** | **Tortuosity (L / D)** | **Curvature proxy (rad / length)** | **Alignment index (0–1)** |
| --- | --- | --- | --- | --- | --- | --- | --- |
| hAC | D7 | 6 | 0 | 0 | NA | NA | NA |
| hAC | D14 | 6 | 2.60 ± 1.82 | 11.18 ± 8.45 | 1.082 ± 0.055 | 0.674 ± 0.127 | 0.530 ± 0.436 |
| hAC | D21 | 6 | 3.10 ± 2.01 | 11.91 ± 9.37 | 1.080 ± 0.036 | 0.871 ± 0.049 | 0.540 ± 0.392 |
| iChon | D7 | 7 | 0 | 0 | NA | NA | NA |
| iChon | D14 | 6 | 1.38 ± 0.97 | 6.83 ± 4.67 | 1.037 ± 0.078 | 0.340 ± 0.108 | 0.54 ± 0.326 |
| iChon | D21 | 10 | 332.20 ± 336.44 | 1910.96 ± 1782.83 | 1.082 ± 0.028 | 0.669 ± 0.063 | 0.217 ± 0.086 |
| hPDC | D7 | 6 | 31.00 ± 16.09 | 228.38 ± 120.86 | 1.039 ± 0.019 | 0.461 ± 0.074 | 0.406 ± 0.098 |
| hPDC | D14 | 9 | 296.33 ± 130.45 | 2006.60 ± 794.55 | 1.080 ± 0.019 | 0.618 ± 0.075 | 0.248 ± 0.079 |
| hPDC | D21 | 6 | 172.00 ± 101.02 | 994.24 ± 578.52 | 1.053 ± 0.017 | 0.589 ± 0.050 | 0.303 ± 0.052 |

**Supplementary Table 2.** Primer sequences used for RT-qPCR analysis

| **Gene** | **Forward Primer (5'→3')** | **Reverse Primer (5'→3')** |
| --- | --- | --- |
| *SOX9* | TGGAGACTTCTGAACGAGAGC | CGTTCTTCACCGACTTCCTC |
| *ACAN* | GTCTCACTGCCCAACTAC | GGAACACGATGCCTTTCAC |
| *COL2A1* | AGATGGCTGGAGGATTTGAT | CTTGCCCCACTTACCAGTGT |
| *COL10A1* | ACGATACCAAATGCCCACAG | GTGGACCAGGAGTACCTTGC |
| *RUNX2* | CGCATTCCTCATCCCAGTAT | GCCTGGGGTCTGTAATCTGA |
| *OSX* | AGTGACCTTTCAGCCTCCAA | GGGAAAAGGGAGGGTAATCA |
| *COL1A1* | GACGAAGACATCCCACCAAT | AGATCACGTCATCGCACAAC |
| *BMP2* | ACTACCAGAAACGAGTGGGAA | GCATCTGTTCTCGGAAAACCT |
| *β-ACT* | CCCAGATCATGTTTGAGACCT | CCTCGTAGATGGGCACAGT |

**Supplementary Table 3.** ICRS macroscopic evaluation of cartilage repair

| **Cartilage repair assessment ICRS** | **Points** |
| --- | --- |
| **I. Degree of defect repair** |  |
| In level with surrounding cartilage | 4 |
| 75% repair of defect depth | 3 |
| 50% repair of defect depth | 2 |
| 25% repair of defect depth | 1 |
| 0% repair of defect depth | 0 |
| **II. Integration to border zone** |  |
| Complete integration with surrounding cartilage | 4 |
| Demarcating border < 1 mm | 3 |
| 3/4th of graft integrated, 1/4th with a notable border > 1 mm width | 2 |
| 1/2 of graft integrated with surrounding cartilage, | 1 |
| 1/2 with a notable border > 1 mm | 1 |
| From no contact to 1/4th of graft integrated with surrounding cartilage | 0 |
| **III. Macroscopic appearance** |  |
| Intact smooth surface | 4 |
| Fibrillated surface | 3 |
| Small, scattered fissures or cracks | 2 |
| Several, small or few but large fissures | 1 |
| Total degeneration of grafted area | 0 |

**Supplementary Table 4.** Modified O'Driscoll histologic scoring of osteochondral defect repair

| **Parameters** | **Points** |
| --- | --- |
| **I. Cell Morphology** |  |
| Hyaline cartilage-like chondrocytes | 4 |
| Predominantly chondrocytes | 3 |
| Mixed fibroblasts and chondrocytes | 2 |
| Predominantly fibroblasts | 1 |
| No chondrocytes present | 0 |
| **II. Matrix Staining** |  |
| Intense staining, similar to native cartilage | 4 |
| Strong staining | 3 |
| Moderate staining | 2 |
| Weak staining | 1 |
| No staining | 0 |
| **III. Surface Regularity** |  |
| Completely smooth, native-like surface | 4 |
| Mostly smooth | 3 |
| Moderately irregular surface | 2 |
| Irregular surface | 1 |
| Severe fibrillation | 0 |
| **IV. Structural Integrity** |  |
| Completely continuous, native-like tissue | 4 |
| Mostly continuous | 3 |
| Some areas of discontinuity | 2 |
| Partial tissue integration | 1 |
| No tissue integrity | 0 |
| **V. Thickness** |  |
| 100%+ native-like thickness | 4 |
| 80-100% of native thickness | 3 |
| 50-80% of native thickness | 2 |
| 20-50% of native thickness | 1 |
| <20% of native thickness | 0 |
| **VI. Integration with Adjacent Tissue** |  |
| Completely integrated | 4 |
| Mostly integrated | 3 |
| Partial integration | 2 |
| Weak integration | 1 |
| No integration | 0 |
| **VII. Subchondral Bone Formation** |  |
| Fully restored subchondral bone | 4 |
| Near-normal bone formation | 3 |
| Moderate bone formation | 2 |
| Minimal bone formation | 1 |
| No subchondral bone | 0 |
| **VIII. Chondrocyte Clustering** |  |
| <10% clustering (similar to native tissue) | 4 |
| 10-25% clustering | 3 |
| 25-50% clustering | 2 |
| 50-75% clustering | 1 |
| >75% clustering | 0 |
